# Role of Plant-Derived Antioxidants in Oxidative Stress-Associated Myocardial Infarction: Structure–Activity Relationship (SAR)-Based Mechanistic Insights

**DOI:** 10.3390/molecules31142506

**Published:** 2026-07-17

**Authors:** Md. Ashraful Alam, Asma Aktar, Ayesha Begum, Md. Liakot Ali, Fariha Sultana Etu, S. M. Naim Uddin, Koichi Fukase, Mohammed Kamrul Hossain, Kishor Mazumder

**Affiliations:** 1 Department of Pharmacy, Jashore University of Science and Technology, Jashore 7408, Bangladesh; a.alam01@just.edu.bd (M.A.A.); a.aktar@just.edu.bd (A.A.); farihaetu304@gmail.com (F.S.E.); 2 Department of Pharmacy, University of Chittagong, Chittagong 4331, Bangladesh; ayeshabegum1749@gmail.com (A.B.); liakotpranto@gmail.com (M.L.A.); pharma.naim@cu.ac.bd (S.M.N.U.); 3 Institute for Radiation Sciences, Graduate School of Sciences, The Osaka University, Toyonaka-shi 560-0043, Osaka-fu, Japan; koichi@chem.sci.osaka-u.ac.jp; 4 School of Optometry and Vision Science, University of New South Wales, Sydney 2052, Australia

**Keywords:** myocardial infarction, reactive oxygen species, oxidative stress, antioxidant, phytochemicals, structure–activity relationship

## Abstract

Among cardiovascular diseases, myocardial infarction (MI) has become one of the leading causes of mortality worldwide, and the prevalence is anticipated to rise considerably in the coming years. Within non-surgical procedures, chemical drugs, including diuretics, vasodilators, calcium channel blockers, ꞵ blockers, angiotensin converting enzyme inhibitors, are now a well-established option to treat MI progression. However, these drugs are not developed to mitigate oxidative stress directly, which has been recently proven to contribute to MI advancement. Naturally occurring antioxidant compounds possess promising cardioprotective properties and have the potential to be used both as lead compounds for finding novel drugs and complementary therapy to manage MI. While some of them, namely quercetin, puerarin, α-lipoic acid, and curcumin, have already made their way up to clinical trials, numerous compounds have not been sufficiently investigated clinically. To develop and formulate natural antioxidant compounds as drugs against MI, it is crucial to comprehend their underlying mechanisms of cardio-protective activities and structure–activity relationships (SARs). This comprehensive review sheds light on the contribution of oxidative stress in the pathogenesis and progression of Myocardial Infarction, and highlights the cardio-protective roles of 51 natural antioxidant compounds along with their mechanistic insights and SAR.

## 1. Introduction

Myocardial infarction (MI) is a common clinical condition in cardiovascular diseases (CVDs) affecting cardiac tissues and ultimately leading to their necrosis due to blood perfusion arrest, failure in supplying oxygen and nutrients, and toxic substance accumulation [1,2]. It is categorized into distinct classes based on pathological, clinical, and prognostic characteristics, as well as treatment options, such as Type 1, Type 2, Type 3, Type 4, and Type 5 [3]. Type 1 MI results from atherothrombotic coronary artery disease (CAD) [3], whereas Type 2 myocardial infarction (T2MI) occurs when the heart’s demand for oxygen or supply of oxygen rises without a sudden disruption in atherothrombotic plaque [4]. In type 2 myocardial infarction, the metabolic needs of myocardial cells are also greater than the amount of oxygen in the blood [5]. Although Type 3 MI is rare (as reflected by a study that found an annual incidence below 10 cases per 100,000 persons and a frequency of 3% to 4% among all MI types), it frequently causes sudden death even prior to obtaining biomarkers or electrocardiogram (ECG) confirmation [3,6]. Among other types, Type 4 MI results from PCI (percutaneous coronary intervention) procedure and thrombosis of a coronary stent, while Type 5 MI is caused by coronary artery bypass grafting (CABG) [6].

A key pathological hallmark of MI is oxidative stress, which describes a condition in which there is a mismatch between oxidants and antioxidants in the body [7]. Oxidative stress is caused by an imbalance between the production of reactive oxygen and nitrogen species (ROS/RNS) and the amounts of enzymatic and non-enzymatic antioxidants. ROS and RNS are the two primary classes of free radicals involved in cellular redox signaling processes [8]. While the ROS encompass superoxide anion, hydroxyl radicals, and hydrogen peroxide, members of the RNS family include peroxynitrite and nitrosoperoxycarbonate [9]. Even though ROS play physiological roles possibly by controlling cardiomyocyte survival and death, their uncontrolled abundance deteriorates cardiac pathological conditions by inducing oxidative stress [10].

To defend against oxidative stress, cells depend mostly on antioxidant enzymes and non-enzymatic antioxidants, among others [11,12]. Antioxidant enzymes, also termed as metalloproteins, fall under the class of proteins that catalyze the conversion of reactive oxygen species (ROS) and possibly their byproducts into more stable, generally less hazardous species. In pathological conditions like MI, where endogenous defenses are inadequate, there is a therapeutic need for exogenous antioxidants, and plant-produced natural antioxidants, including flavonoids, phenolics, and polyphenolics (hydrolysable and condensed tannins), have this therapeutic potential as they have been shown to prevent reactive oxygen species-induced oxidative stress [3]. These natural bioactive compounds may lower oxidative stress in humans by getting rid of free radicals, slowing down lipid peroxidation, and stopping nitrosation reactions [13]. In order to eliminate the free radicals that are produced as a byproduct of oxidative metabolism and to keep the redox balance in its proper place, natural antioxidants can work either independently or in conjunction with one another. Although current medications for treating MI are well-established and standard clinical guideline-supported, they are not developed to reduce oxidative stress directly, which could be another possible investigational approach in managing MI. Natural bioactive antioxidant compounds possess promising cardioprotective effects and have the ability to mitigate oxidative stress associated with MI progression via several mechanisms. Therefore, scientific interest has grown in these compounds for developing novel drugs and finding possible complementary or adjunct therapies for MI.

To enhance the understanding and optimization of these natural antioxidant compounds, it is imperative to study their SAR. SAR correlates the structural features of the molecule to its therapeutic activity. For example, the quantity and location of hydroxyl groups in flavonoids predict their ability to scavenge radicals, and the conjugated double bonds in carotenoids enable them to be better at quenching singlet oxygen. Moreover, during the journey of drug discovery, phytochemicals have to undergo critical scrutiny to be developed as valid therapeutic agents for MI. Such scrutiny looks at their structural attributes, SAR, and underlying mechanism of action to make sure that they may be considered as potential candidates in developing drug molecules. Although there are mounting scientific data regarding the cardio-protective activity of these natural bioactive compounds, there is still a dearth of systematic investigations that link SARs to their cardio-protective action pathway in MI. To address this gap, this review aims to provide a concise summary of the role of oxidative stress in the development and progression of MI, highlight the identified natural antioxidant compounds that alleviate MI and its associated complications, and finally explore the SAR of some of the most promising compounds to understand how their structural features contribute to their cardio-protective activity.

## 2. Method

**Figure 1:**
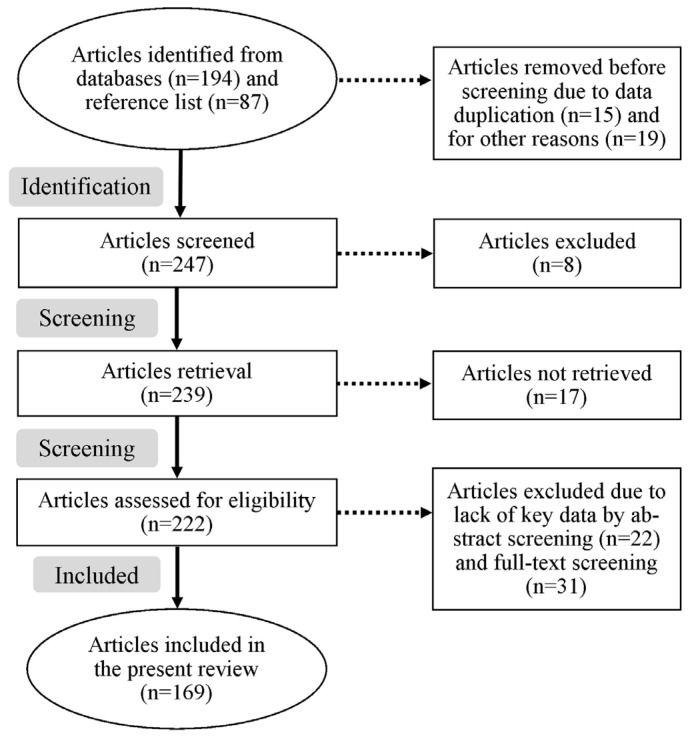
PRISMA diagram showing the study selection procedure.

In order to garner the relevant literature, well-known scientific databases, including Google Scholar, Springer Link, Science Direct, PubMed, Scopus, Web of Science, and the Wiley Online Library, were accessed from February 2024 to May 2026. Both the recent and older classic articles (1993–2026) were critically analyzed and reviewed in this study. While selecting relevant articles for inclusion, those studies were prioritized that were published in reputed journals indexed by internationally recognized databases and journal ranking systems such as SCImago Journal Rank. The search procedure was led by writing specific keywords such as “myocardial infarction”, “oxidative stress in myocardial infarction”, “natural antioxidant compounds”, “structure–activity relationship”, and “myocardial infarction mechanism”. These keywords were combined with each other by utilizing Boolean operators (“AND” and “OR”) to generate various search combinations during the search procedure. Only publications published in the English language were considered, as articles in other languages could not be evaluated due to linguistic constraints. We excluded the articles that were duplicates, unpublished, irrelevant, or lacked detailed information. This review covered research studies on the following topics: association of oxidative stress with myocardial infarction, plant-derived anti-oxidant compounds with cardio-protective effects to alleviate MI, along with their underlying mechanisms of effects, and their SAR that contributes to these activities. The detailed study selection procedure is demonstrated through a PRISMA diagram in Figure 1.

## 3. Oxidative Stress in MI Development

Free radicals are regarded as reactive chemical species that possess a single unpaired electron in the outermost orbital [14,15]. They are capable of either donating or accepting electron species, therefore can act as both oxidant and reductant [16,17]. The majority of free radicals that are engaged in impairing the biological system are known as oxygen-free radicals; more particularly, “reactive oxygen species” (ROS). These ROS cause myocardial damage during ischemia or reperfusion [18]. Overproduction or excessive accumulation of free radicals alters homeostasis, which subsequently gives rise to pathological conditions. For example, hydroxyl radical and peroxynitrite in excess can damage cell membranes and lipoproteins by lipid peroxidation that leads to the formation of malondialdehyde (MDA) and conjugated diene compounds, which are mutagenic and cytotoxic.

Oxidative stress occurs due to the imbalance between free radical generation and antioxidant defensive mechanisms. In other words, the term “oxidative stress” refers to the disruption between reduced to oxidized glutathione or (GSH/GSSG) or NADPH/NADP+ ratios [19]. It can be a consequence of either—due to the impairment of the antioxidant system caused by malnutrition (inadequate consumption of vitamin C, vitamin E, sulfur-containing amino acids) or by the excessive production of free radicals, including ROS and RNS (due to external or internal stimulus). Cells can bear up mild oxidative stress because there is an upregulation of the antioxidant system in response to that mild rise, so that cells can cope with the raised situation. For example, if experimental rats are continuously exposed to higher levels of oxygen, they develop better oxygen tolerance in comparison to control rats due to an elevation in the antioxidant system in the lungs [20]. But when this adaptive capacity of the cell exceeds, it starts to experience oxidative stress. During the development and advancement of MI, the surge of oxidative stress may cause mitochondrial dysfunction and ferroptosis. Ferroptosis is a type of iron-dependent regulated cell death. It occurs because of the oxidative stress damaging the polyunsaturated fatty acids of the cell membrane, a process known as lipid peroxidation. As a result of mitochondrial dysfunction, ROS are produced in great numbers due to the impairment of the electron transport chain. These excessive ROS lead to further oxidative stress-induced lipid peroxidation, which eventually results in myocardial cell injury and cell death through ferroptosis [21,22].

### 3.1. Pathophysiology of Oxidative Stress-Associated MI

**Figure 2:**
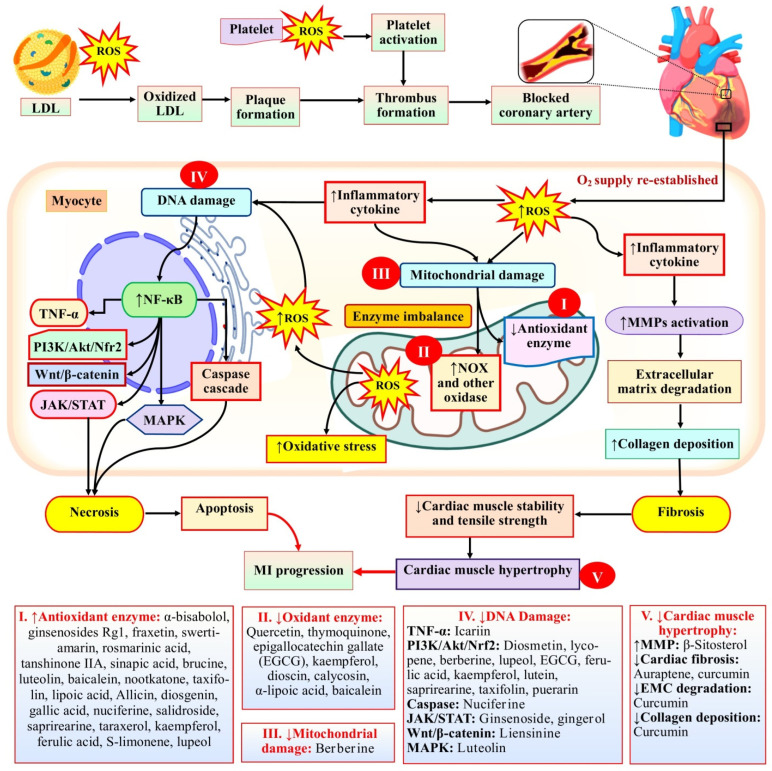
Impact of natural compounds on alleviating oxidative stress-associated MI progression along with their corresponding intervention points.

The multifaceted roles of oxidative stress in MI pathogenesis are summarized and presented in Figure 2. MI occurs in a series of events after the supply of oxygen in cardiovascular tissue is acutely hampered, mostly due to coronary thrombosis resulting from atherosclerotic plaque rupture and platelet activation, a condition known as ischemia [23]. This shifts cellular respiration to anerobic, and also increases intracellular calcium level that results in cell swelling, rupture, and mostly cell death by both necrosis and apoptosis [2,24]. When the supply of oxygen to tissue is reestablished, reactive oxygen species are produced in cardiac muscle cells that contribute to the prognosis of this disease. Mitochondria, as well as some other cardiovascular enzymes such as NADPH oxidase (NOX), nitric oxide synthase (when uncoupled), and xanthine oxidase, are responsible for the generation of ROS. Excessive generation of ROS leads to oxidative stress-mediated impairment of mitochondrial functions. Consequently, even more ROS are produced, increasing the level of inflammatory cytokines, which in turn induce further production of ROS and damage vital cellular structures, including mitochondria. This disastrous loop of mitochondrial failure, increased oxidative stress, and cellular destruction keeps ramping up and eventually expedites MI progression [25,26]. Moreover, ROS and inflammatory cytokines trigger the increased activation of matrix metalloproteins (MMPs) and excessive deposition of collagen. MMPs are enzymes responsible for the degradation of the extracellular matrix components (ECMs), including collagen, which offers cardiac muscle its structural stability and tensile strength. Even though in normal circumstances MMPs have a crucial role in tissue remodeling, ROS and inflammatory cytokines mediated overactivation of them during the initial stage of MI speeds up ECMs and collagen breakdown, leading to diminishing strength of cardiac muscle. This is succeeded by an excess accumulation of collagen, a condition referred to as fibrosis, which leads to an increase in the stiffness of the cardiac muscle. Both processes aid in the alterations of the ventricular remodeling of injured myocardium following MI. ROS can also directly initiate cascades of intracellular signals that exacerbate cardiac damage. For example, hydrogen peroxide directly stimulates the production of tumor necrosis factor α (TNF-α) via the p38 mitogen-activated protein kinase (MAPK) pathway that mediates myocardial dysfunction and apoptosis [2,27]. Likewise, in experimental rat models of heart failure, the superoxide anion in the aorta was observed to be markedly increased in the same p38 MAPK pathway in addition to the elevated level of the NOX subunit p47phox [28].

Furthermore, in post-myocardial infarction patients, a consistently high level of hydroxyl radical production was reported to be associated with the likelihood of progressing towards heart failure according to a clinical study [29]. These aforementioned oxidizing species can further oxidize LDL, leading to a rise in oxidized LDL or oxLDL in the circulation. Elevated concentration of circulating oxLDL is linked with various cardiovascular diseases, including atherosclerosis [30,31,32]. Apart from inflicting direct damage, ROS also function as secondary messengers in cardiac signaling pathways. For instance, Src family of tyrosine kinases, MAPKs, small GTP-binding proteins, and cytokines are all activated by ROS. These subsequently induce the activation of nuclear transcription factors connected to the cellular stress responses, which eventually result in cell hypertrophy and apoptosis [33].

### 3.2. Impact of Oxidative Stress on MI Complications

MI leads to a number of complications, which include cardiogenic shock, ventricular remodeling, left ventricular free wall rupture, and heart failure, among others [34,35,36,37,38,39]. Oxidative stress has a strong impact on MI complications. In myocardial reperfusion injury, the restoration of blood flow to the already existing ischemic zone causes a significant increase in ROS, which quickly and severely damages crucial bio-molecular constituents of the myocardium. Thus, the myocardium loses its structural integrity and functionality to a great extent. This phenomenon is accompanied by several post-MI complications, including lethal reperfusion, no-reflow phenomenon, myocardial stunning, and reperfusion arrhythmias. Post-MI patients who also have diabetes mellitus experience worsened oxidative stress conditions [40]. It is because the constant presence of hyperglycemia is reported to promote oxidative stress succeeding MI, causing endothelial dysfunction and the advancement of atherosclerosis [41]. This obstructs the coronary supply of oxygenated blood to the myocardium, thereby further deteriorating the condition of heart cells. So, together, diabetes and myocardial ischemia induce oxidative stress, which causes cardiac tissue to overexpress iNOS and increase NO production. Inhibited NADPH activity may interact with the latter, causing the generation of peroxynitrites and a decrease in NO bioavailability. These responses are closely tied to the rise in cytokine production and apoptosis inside the myocardium, which increases necrosis and disrupts the post-MI recovery of cardiac cells [42].

## 4. Biomarkers of Oxidative Stress in MI

There are numerous biomarkers of oxidative stress that are present in patients with MI, indicating an association between oxidative stress and MI (Table 1). For example, according to a study by Feng et al., MI patients have higher levels of advanced oxidation protein products (AOPP) in their blood [43]. Protein carbonyl is a significant biomarker for protein oxidation and it was also found significantly higher in patients with MI than in controls, indicating the ROS-driven oxidative protein damage as a result of MI [44].

It is worthwhile to mention that ROS causes the oxidation of LDL, which raises the levels of circulating oxidized LDL (oxLDL), a marker that has been linked to the emergence of CVDs. oxLDL stimulates the growth and accumulation of foam cells in atherosclerotic plaques, which may then favor platelet activation and cause a cycle of oxidative damage. Also, lipid peroxidation products such as MDA, HNE, and F2-isoprostanes, which are derived from polyunsaturated arachidonic acid, are important biomarkers for oxidative stress linked to cardiovascular complications [45]. Several studies found that increased levels of MDA and F2-isoprostanes are associated with exacerbation of MI [46,47,48,49,50].

Several vitamins, such as vitamin A, C, and E, act as antioxidants and protect the body from harm by neutralizing free radicals. A study revealed that patients with acute MI who were hospitalized to intensive care had significantly lower levels of vitamins E and C, except for vitamin A, which did not differ significantly [51]. Another study reported that individuals with AMI had relatively lower levels of vitamins E and C than controls [52]. This is in line with the findings of Singh et al., who reported that lipid peroxide levels were much greater in AMI patients compared to controls, whereas vitamins C, E, A, and β-carotene decreased dramatically [53].

In different studies, patients with MI and ischemic heart disease were observed to have considerably lower superoxide dismutase (SOD), catalase (CAT), and glutathione peroxidase (GPx) activity than healthy controls [54,55]. Additionally, smokers with AMI had significantly lower SOD, CAT, and GPx activity than nonsmokers, making these people more susceptible to oxidative stress [56]. SOD and CAT activity in the erythrocytes of AMI patients has been decreased due to elevated peroxidation [57,58]. In the erythrocytes of AMI patients, there has been evidence of reduced GPx activity. Moreover, individuals with AMI have considerably lower GSH concentrations in plasma and erythrocytes [59,60,61]. So, levels of SOD, CAT, GPx, and GSH are decreased in MI due to oxidative stress, which was confirmed by several studies [44,62,63,64]. The amount of zinc, magnesium, and calcium in the body is related to oxidative stress. MI patients had significantly lower serum zinc levels within the first three days than the control group. In MI patients, Zn may therefore be a helpful oxidative marker [65]. A lack of magnesium also increases the formation of free radicals linked to MI [47]. It is noteworthy to mention that Mg exhibits antiarrhythmic properties in MI. Acute MI is more likely to be fatal and is related to low serum Mg levels. Mg shortage worsens MI through increasing oxidative stress-induced ischemia damage and mitochondrial dysfunction [66].

While several oxidative stress indicators change after myocardial infarction, there are significant differences in their clinical value. Biomarkers like MPO and oxLDL have demonstrated potential as supplemental indicators for early diagnosis and risk classification, because they indicate oxidative damage and inflammatory activity linked to plaque instability and cardiac damage [67,68]. MDA, HNE, and F2-isoprostanes are examples of lipid peroxidation products that are more frequently utilized to assess the degree of oxidative damage and may function as prognostic or treatment-monitoring markers rather than independent diagnostic instruments. On the other hand, antioxidant vitamins and antioxidant enzymes (SOD, CAT, GPx, and GSH) are commonly used as research biomarkers to evaluate oxidative stress state and therapeutic responses in experimental and clinical trials. They mainly represent the endogenous antioxidant defense system [69,70]. However, due to issues with specificity, standardization, and clinical validation, none of these oxidative stress biomarkers has yet been used in routine clinical practice to replace well-known cardiac biomarkers like cardiac troponins.

The lack of specificity of oxidative stress indicators for myocardial infarction is a significant drawback. In addition to MI, a variety of clinical and physiological situations affect several biomarkers, including MDA, AOPP, protein carbonyls, oxLDL, MPO, F2-isoprostanes, and antioxidant enzyme activity. Biomarker levels may also be influenced by other factors, including comorbid diseases (diabetes mellitus, chronic renal disease, systemic inflammation, hypertension, and other cardiovascular conditions), smoking, renal function, drugs (such as statins and antioxidant supplements), dietary antioxidant intake, and lifestyle variables [71,72,73,74]. Therefore, rather than being employed as stand-alone diagnostic indicators, these biomarkers should be interpreted carefully and ideally in conjunction with established cardiac biomarkers, clinical presentation, electrocardiographic results, and imaging tests.

**Table 1:** Biomarkers of oxidative stress in myocardial infarction.

Biomarker	Role in MI Pathophysiology	Reference
Advanced oxidation protein products (AOPPs)	AOPPs, indicative of extensively oxidized proteins, are significantly elevated in AMI patients experiencing acute hyperglycemia. These pro-inflammatory mediators directly disrupt HDL metabolism and may play a crucial role in the progression of cardiovascular disease.	[43]
Protein carbonyl (PC) content	PC content, a widely recognized marker of protein oxidation, is found to be elevated in AMI patients, suggesting protein damage as a result of AMI.	[75]
Oxidized LDL (oxLDL)	oxLDL promotes the proliferation and buildup of foam cells within atherosclerotic plaques, potentially triggering platelet activation and perpetuating a cycle of oxidative damage.	[76]
Lipid peroxidation products	Lipid peroxidation products (MDA, HNE, and F2-isoprostanes) derived from arachidonic acid serve as biomarkers of oxidative stress related to CVD. Increased levels of MDA and F2-isoprostanes are linked to the worsening of MI.	[77]
SOD, CAT, and GPx	In MI or IHD patients, SOD, CAT, and GPx levels are significantly lower compared to healthy subjects.	[64]
Antioxidant vitamins	AMI patients have notably lower levels of vitamins C, E, A, and β-carotene compared to the control group.	[53,78]
Zinc and magnesium	Low serum zinc levels are observed during acute tissue injury such as MI. Similarly, magnesium deficiency increases ROS generation associated with MI. Magnesium usually plays a preventive role in MI. Hence, lower serum Mg levels are linked to a higher fatality risk in AMI.	[66,79]

## 5. Natural Antioxidant Products-Based Therapeutic Approaches for MI

**Figure 3:**
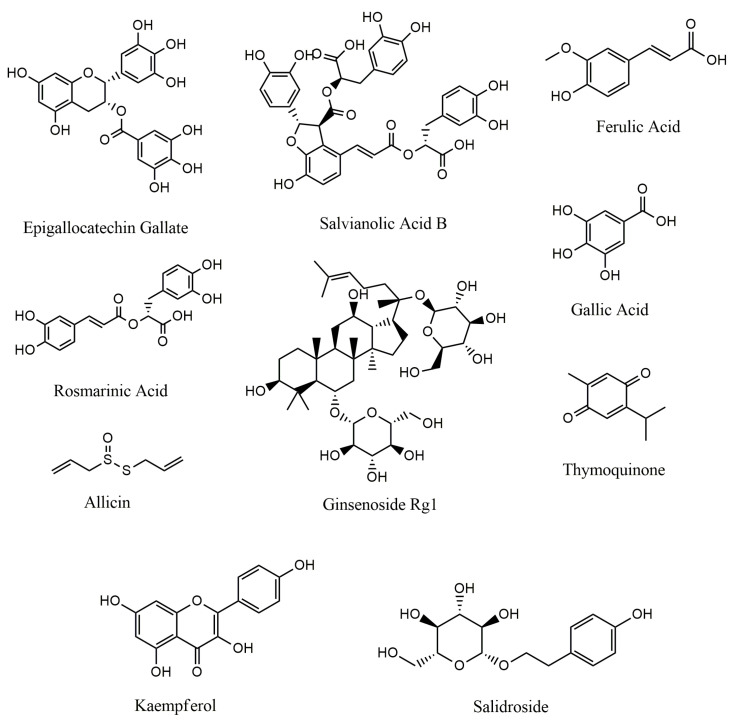
Representative chemical structures of antioxidant compounds.

In recent years, scientists have realized how crucial oxidative stress is to the emergence of MI [80]. Excessive oxidative stress may be a factor in the ischemia/reperfusion injury, which accounts for MI-related mortality. This revelation prompts researchers to explore innovative therapeutic approaches that specifically target this particular path in the context of anti-MI drugs [81]. Considering this, in this section, we have provided an overview of bioactive natural compounds reported for potential MI management, focusing on mitigating oxidative stress. Representative chemical structures of antioxidant compounds are given in Figure 3. Compounds in Figure 3 are chosen due to their extensive preclinical evidence in MI and diverse chemical structures. A summary of reported natural bioactive compounds along with their mechanistic insights into cardioprotection in MI, physicochemical properties, typical experimental dose and pharmacokinetic limitations is also presented in Table 2.

### 5.1. Flavonoids

#### 5.1.1. Quercetin

Quercetin is a potent flavonoid commonly found in wide varieties of plants. It is also very commonly found in vegetables, fruits, berries, and teas. This compound is reported to increase the level of nitric oxide in the ischemic myocytes of the myocardium [82]. It also exhibits the potential to inhibit several oxidative enzymes, like oxidases and lipoxygenases. More recently, it is known as a potent antioxidant medication [83,84]. A recent study conducted by Kozhukhov et al. has revealed that quercetin aids in protecting the heart by reducing the infarct size and stopping intramyocardial hemorrhage when administered as intravenous infusion alongside the conventional treatment in patients following the first incidence of ST elevation MI [85]. However, quercetin was also reported to have low oral bioavailability, poor water solubility, gastrointestinal degradation, and rapid phase II metabolism [86,87].

#### 5.1.2. Formononetin

It has been widely reported that the isoflavone formononetin (FN), which is found in many plants but is particularly abundant in *Trifolium pratense* (red clover) and the Chinese herbal supplement *Astragalus membranaceus*, has a variety of pharmacological properties, including antioxidant, cardioprotective and neuroprotective activity, vasorelaxant, anticancer, anti-inflammatory, and antiviral activity. Wang et al. designed an experimental investigation to better understand how FN affects myocardial ischemia/reperfusion injury (MIRI) [88]. The results demonstrated that FN alleviated MIRI in rats and reduced the activation of the NLRP3 inflammasome through the regulation of the ROS-TXNIP-NLRP3 pathway.

#### 5.1.3. Epigallocatechin Gallate

Epigallocatechin gallate (EGCG) is a naturally occurring flavonoid and one of the main catechins found in green tea. It is recognized for its potent antioxidant and cardioprotective properties. Studies in H9C2 cardiomyocytes and male C57BL/6 mice have demonstrated that EGCG at a dose of 50 mg/kg effectively alleviates myocardial ischemia–reperfusion injury by reducing oxidative stress and inhibiting cardiomyocyte apoptosis. Treatment with EGCG has been seen to significantly decrease MDA levels and Bax expression, along with enhancing SOD activity and Bcl-2 overexpression. In addition, it modulates the PI3K/Akt signaling pathway and exhibits antihypertensive effects through down-regulation of vascular NADPH oxidase activity, leading to improved vascular function. Apart from these effects, its clinical application is hindered by low bioavailability, poor chemical stability, and rapid systemic clearance. Advanced delivery systems such as nanoparticle-based delivery are being investigated to enhance their pharmacokinetic properties and therapeutic efficacy in cardiovascular diseases [89,90,91].

#### 5.1.4. Baicalein

Baicalein, the primary bioactive component found in the roots of *Scutellaria baicalensis* and *Scutellaria lateriflora*, has a wide range of pharmacological activities, including anti-inflammatory, anti-cancer, anxiolytic, antidepressant, and antioxidant action. Kumar et al. assessed the effects of baicalein pretreatment on ISO-induced MI and looked into potential causes [92]. The results of their investigation suggest that pretreatment with baicalein protects the rat myocardium from ISO-induced myocardial infarction. Additionally, Baicalein preserves the cardiac enzyme functioning, sustains the integrity of the myocardium, inhibits oxidative–nitrosative stress, boosts the antioxidant defense system, and suppresses NF-κB protein production, which minimizes necrosis and/or cell death in rats with myocardial infarction.

#### 5.1.5. Luteolin

Luteolin is a falconoid substance with anti-inflammatory, antioxidant, and anti-tumor properties. It can be obtained from traditional Chinese remedies. Recently, it has been used in clinical treatment for the prevention of IHD and MIRI. Luteolin markedly reduces the elevated ROS generation due to its ability to balance out the oxidant/antioxidant system. According to Yu et al., in vivo and in vitro myocardial ischemia/reperfusion has been successfully treated with luteolin [93]. The ROS system and MAPK pathways may be involved in mediating the effect, which improves mitochondrial function by inhibiting the phosphorylation of JNK and p38 MAPK, facilitating the activation of ERK1/2, and increasing the transcription of Mn-SOD’s mRNA.

#### 5.1.6. Icariin

Icariin is the primary bioactive medicinal ingredient identified from *Epimedii herba*. Inhibition of oxidative stress, attenuation of DNA damage, prevention of mitochondrial oxidative damage, and reduction of cardiomyocyte apoptosis are just a few of the many potential pathways that could underlie icariin’s cardioprotective benefits. An in silico study by Ke et al. [94] hypothesized that suppressed oxidative stress may be related to icariin’s protective effects against MI. This protective characteristic makes icariin a candidate for treating MI. However, it should be mentioned that computational predictions sometimes may overlook key biological factors and are associated with experimental errors to a great extent [95].

#### 5.1.7. Calycosin

Calycosin is one of the *Radix astragali*’s main active ingredients, having cardioprotective qualities. In order to figure out if calycosin can reduce oxidative stress and oxidative stress-induced cardiac apoptosis in newborn cardiomyocytes (NCMs) via activation of aldehyde dehydrogenase 2 (ALDH2), calycosin has been studied in vivo and in vitro by Ding et al. [96]. The results support the potential future investigation of calycosin in MI by indicating that it lowers oxidative stress and oxidative stress-induced apoptosis through the control of ALDH2 signaling.

#### 5.1.8. Diosmetin

Diosmetin is a citrus fruit-derived mono-methoxy flavonoid commonly found in lemons and olives. It is also extracted from medicinal herbs like *Rosmarinus officinalis*. Diosmetin is reported to inhibit MDA formation as well as restore antioxidant enzyme functions in the myocardium [97]. In an H9c2 cell line-based study on hypoxia-afflicted damaged myocardium, Si et al. reported that Diosmetin reduces the apoptosis of myocardium via the autophagy induction by activating AMPK [98]. This study suggested Diosmetin as a potential drug candidate in the treatment of myocardial infarction.

#### 5.1.9. Puerarin

Puerarin is the principal isoflavone obtained from *Radix puerariae*. It has anti-inflammatory, anti-oxidative, and anti-apoptotic properties. An experimental model is developed and examined to find out if puerarin pretreatment enhances heart function in AMI [99]. The outcomes demonstrated that pretreatment with puerarin in AMI can significantly enhance cardiac function by preventing myocardial apoptosis. The PI3K/Akt pathway in cardiomyocytes may be activated in order to mediate the molecular mechanism of this protective action. Puerarin also underwent a clinical trial, which is discussed in detail in Section 8 [100].

#### 5.1.10. Kaempferol

Kaempferol is an active phytoconstituent of *Amaranthus viridis*. An ISO-induced rat model was used by Krishna et al. to study the cardioprotective effects of kaempferol [101]. The study reported that kaempferol can improve antioxidant response by reducing myocardial oxidative stress via the Nrf-2/Ho-1 signaling pathway, which is principally responsible for its cardioprotective effects against ISO-induced MI in rats.

#### 5.1.11. Biochanin-A

Biochanin-A (BCA) is an O-methylated isoflavonoid found in plants like red clover, soy, alfalfa sprouts, peanuts, and chickpeas. Govindasami et al. have studied the impact of BCA on MI, which revealed that this compound showed cardioprotection in MI by reducing lipid peroxidation and increasing antioxidant enzyme function [102].

#### 5.1.12. Taxifolin

Taxifolin (TAX) is a naturally occurring flavonoid that is mostly found in olive oil, grapes, and onions. It has a number of pharmacological effects, including anti-inflammatory and radical scavenging capabilities. TAX protects against ISO-induced acute myocardial injury through the activation of the Nrf2/HO-1 signaling pathway, attenuation of oxidative tissue injury, attenuation of key regulators of the inflammatory response and apoptosis. Thus, TAX may be of use to find a novel cardioprotective treatment for acute MI. However, it requires additional investigation in prospective human trials [103].

Out of all the discussed flavonoid compounds, only quercetin and puerarin have preliminary clinical evidence for use against MI, whereas the remaining compounds are supported by preclinical in vivo, in vitro, and in silico studies. Although these flavonoid compounds showed promising cardio-protective function, they are often associated with translational challenges due to their pharmacokinetic limitations, which have been specifically mentioned in Table 2 as well as in Section 6.

### 5.2. Terpenoids

#### 5.2.1. S-Limonene

S-Limonene (SL) is one of the most prevalent monoterpenes discovered in lemon and orange peels. To assess the potential therapeutic use of SL in an isoproterenol-induced MI animal model, an investigation was conducted by Rhana et al. [81]. The findings demonstrated that s-limonene supports cardio-protection against MI injury, most likely via decreasing elevated Ca^2+^ and reducing oxidative stress via CaMKII. This makes it a potentially effective choice for the treatment of MI. An investigation conducted in Malaysia found the presence of 94% limonene in *Citrus × microcarpa* extracted volatile oil. *Citrus × microcarpa* extracted volatile oil was reported for its promising antioxidant and wound healing potential. However, limonene is also known for its low metabolic and plasma stabilities, low bioavailability and tissue distribution [104,105].

#### 5.2.2. Tanshinone IIA

Tanshinone IIA (TIIA) is a natural compound obtained from the roots of *Salvia miltiorrhiza* Bunge. Several studies have shown that TIIA has antioxidant, anti-cancer, and anti-inflammatory properties [106]. TIIA is utilized to treat cardiovascular disorders because it can improve myocardial metabolic dysfunction caused by hypoxia. It can also improve coronary blood flow. It is reported that TIIA therapy can lessen infarction and boost myocardial regeneration and contractility [107]. Moreover, CHEN et al. found that TIIA might alleviate cardiac dysfunction and fibrosis in heart failure (HF) of MI rats by preventing oxidative stress [108]. The imbalance between antioxidant and oxidant levels was also rectified by TIIA.

#### 5.2.3. Nerolidol

Nerolidol is a plant-derived sesquiterpene alcohol commonly found in lavender, lemongrass, and ginger [109]. It is reported to have a wide number of pharmacological activities, like antioxidant, anticancer, antinociceptive, and anti-inflammatory activities [110,111,112]. A recent study has demonstrated its effectiveness in the prevention of myocardial damage when administered for a long time in animal models [113]. Another research study reported the cardioprotective role of nerolidol in MI, where different oxidative stress markers were found to be altered. It significantly increased SOD activity and reduced lipid peroxidation and carbonyl levels, leading to minimizing isoproterenol-induced heart damage [113,114].

#### 5.2.4. Lupeol

A pentacyclic triterpenoid molecule known as lupeol is abundantly present in edible plants like olive oil, strawberries, etc. According to Moris et al. [115], unusual ROS production and NF-κB activation are key determinants of the severity of myocardial damage. Also, Nrf2 has been linked to the control of oxidative stress responses as a key transcription factor. In order to find out whether lupeol has protective benefits against myocardial ischemia/reperfusion injury (I/R) via modulating NF-κB and Nrf2 signaling pathways, Li et al. [116] used myocardial I/R rat models. The results revealed that lupeol dramatically altered NF-κB and Nrf2 signaling, suggesting that it may have potential for preventing I/R-induced myocardial infarction.

#### 5.2.5. Nootkatone

Nootkatone (NKT), a naturally occurring bioactive sesquiterpene that is widely present in grapefruit, is one of many well-known plant-derived substances. It has gained attention for its potential health advantages and pharmacological activities. Meeran et al. looked into NKT’s ability to protect against MI in the hearts of rats [117]. The results showed that, in ISO-induced MI, NKT’s protective actions occur by activating the PI3K/Nrf2/Akt signaling cascades and moderating aberrant TLR4/NF-κB/MAPK signaling, which in turn reduced oxidative stress, inflammation, and apoptosis.

#### 5.2.6. α-Bisabolol

α-Bisabolol is a sesquiterpene alcohol that is present in the essential oils of several different plants, such as chamomile (*Chamomilla recutita* L.), salvia (*Salvia runcinata*), *Plinia cerrocampanensis*, and wood of candeia (*Eremanthus erythropappus*) [118]. It has been demonstrated that bisabolol has anti-inflammatory, antimutagenic, gastroprotective, antispasmodic, and antioxidant activities [119]. As free radical-mediated oxidative stress is crucial for MI development, Meeran et al. carried out an investigation to see how α-Bisabolol affects lipid peroxidation, nonenzymatic antioxidants, and hemodynamics in ISO-induced MI [120]. The in vivo and in vitro hemodynamic and biochemical data were obtained from that study. The study revealed that bisabolol protects the myocardium of ISO-induced MI rat models due to its powerful anti-lipid and antioxidant effects. The reversal of altered lipid peroxidation and nonenzymatic antioxidant status was observed following the treatment with α-Bisabolol.

#### 5.2.7. Bakuchiol

Bakuchiol (BAK) is a bioactive compound extracted from the seeds of the *Psoralea corylifolia* plant. It is known to have preventive effects against cardiac complications due to its antioxidant and anti-inflammatory capabilities. BAK has shown cardio-protective activity by alleviating hyperglycemia-induced diabetic cardiomyopathy, improving aortic banding-induced pathological cardiac hypertrophy, and attenuating MIRI-induced mitochondrial oxidative damage in isolated heart cells. BAK maintains cardiac function following MI and also protects the heart from harmful remodeling. It most likely acts by inhibiting the gene expression of ERK2 and TGF-β1 [121].

#### 5.2.8. Saprirearine

Saprirearine is a diterpenoid derived from *Salvia prionitis*. Recent research on this compound explored that it possesses cardioprotective activity on hypoxic/re-oxygenated H9c2 cardiomyocytes model of MI. The underlying mechanism involves enhancing mitochondrial dysfunction and preventing oxidative stress, thereby preventing mitochondria-mediated cell death. Apart from that, it also activates Nrf2, which synergistically fosters cardioprotection [122].

#### 5.2.9. Taraxerol

Taraxerol is a plant derived naturally occurring pentacyclic triterpenoid with diverse medicinal properties. Recent studies investigated its possible cardioprotective activity to prevent MI, which revealed that it increases antioxidant enzymes like SOD and reduces pro-inflammatory cytokines, leading to protecting the heart from ISO-induced damage [123].

#### 5.2.10. Hinokitiol (β-Thujaplicin)

Hinokitiol (β-thujaplicin), a plant-derived biocompound resembling tropolone, obtained from cupressaceous plants’ wood, exhibits bioactivities like antibacterial, anticancer, anti-inflammatory, and antioxidant activities [124]. Research done by Xiao et al. stated that this compound reduces apoptosis by down-regulating autophagy flux [125].

#### 5.2.11. Thymoquinone

Thymoquinone (TQ) is the primary active component of *Nigella sativa*, giving *Nigella sativa* its anti-inflammatory and antioxidant potentials. To further understand how TQ affects oxidative stress, inflammation, and apoptosis, as well as how it protects cardiac mtDNA in an experimentally induced MI model, an experimental study was conducted by Khalifa, Rashad and El-Hadidy [126]. The finding demonstrates that pre- and co-administration of TQ reduced the oxidative stress linked to the occurrence of MI, reversed the changes in histoarchitecture, and retained the amount of cardiac mtDNA. These characteristics enhance the cardiac advantages resulting from using TQ as a natural cardio-protective agent.

It is noteworthy that only preclinical or in vitro studies have been conducted for all the terpenoid compounds mentioned here, and the clinical translation of terpenoids is reported to be hindered by several factors, including their limited intestinal permeability, low aqueous solubility, rapid elimination, and non-specificity in distribution [118]. However, delivering them using nano-formulation-based drug delivery systems has been reported to help overcome these limitations to a certain extent.

### 5.3. Phenolics

#### 5.3.1. Curcumin

Curcumin is a polyphenolic compound commonly found in turmeric. It is reported to prevent myocardial injury by reducing oxidative stress, inflammation, apoptosis, and fibrosis. Pretreatment with curcumin alleviates MIRI-induced mitochondrial oxidative damage [127]. Xiao et al. reported that curcumin prevents myocardial fibrosis most possibly via the activation of SIRT1 [128]. Another preclinical investigation involving MI rat models revealed the cardiac repairing function of curcumin following MI. Cardiac dysfunction was also mitigated after oral consumption of curcumin [129]. Therefore, it can be used alongside conventional therapy for the better management of myocardial fibrosis following MI. Having stated that, curcumin was also observed to have extremely low oral bioavailability, limited gastrointestinal absorption, and rapid metabolism [130]. Hence, the use of advanced drug delivery systems is recommended to overcome these problems. For example, in a study on ISO induced MI rat models, the impact of curcumin nanoparticles was seen in comparison to traditional curcumin. The study found that nanoparticle formulation was more effective than traditional curcumin in declining the levels of inflammatory parameters, including TNF-α, IL-6, and IL-1α, following MI. In addition to MMP-9 and MMP-2, other cytokines, namely, RANTES, IL-1β, and MCP-1, were also reduced more in nanoparticle form. Likewise, upon histo-pathological assessment, the nanoparticle formulation was reported to be better performing in preventing myocardial necrosis [131].

#### 5.3.2. Ellagic Acid

Ellagic acid (EA) is an antioxidant and anti-inflammatory polyphenol that is mostly present in pomegranates, blackberries, raspberries, strawberries, cranberries, walnuts, pecans, and wolfberries [132]. Plenty of studies reported that EA treatment prevented lipid peroxidation [133], oxidant-induced endothelial dysfunction, and atherosclerosis [134], in addition to inhibiting the isoproterenol-induced cardiac necrosis in rats [135]. Additionally, EA improved the structural and functional recovery of myocardial energy metabolism when paired with indapamide, a thiazide diuretic prescribed for hypertension. Moreover, oral administration of EA reduces ROS production and blocks the CaMKII signaling pathway, which improves left ventricular diastolic dysfunction in rats with ovariectomies. Therefore, EA seems like a viable option that can be further investigated for the treatment of clinical conditions like hypertension, heart failure, and MI [132].

#### 5.3.3. Salidroside (SAL)

SAL has several pharmacological properties that boost immunity, improve tolerance to hypoxia, and reduce free radical-induced damage. A thorough investigation by Fan et al. into SAL’s ability to prevent harm has revealed that it can act in a variety of ways to fight oxidation, inflammation, and apoptosis [136]. In that study, SAL greatly improved the remodeling process of MI and decreased cardiac damage by reducing oxidative and inflammatory effects via the PI3K/AKT/Nrf2/HO-1 pathway. This suggests that SAL has the potential to be investigated from a clinical point of view to treat MI.

#### 5.3.4. Rosmarinic Acid

Rosmarinic acid (RA) is a bioactive phytochemical isolated from the *Rosmarinus officinalis* plant. It has particularly drawn scientific interest due to its antioxidant, anti-inflammatory, anti-depressive, and anti-proliferative properties. RA is able to treat MI-associated depression because it has multidimensional pharmacological effects. Verma et al. investigated the possible benefits of RA on comorbidly depressed MI rat models [137]. The study was designed so that depression (maternal stress) affected the severity of MI. The study divulged that the anti-inflammatory, anti-oxidative, anti-depressive, and brain-derived neurotrophic factor modulatory capabilities of RA provide cardio-protection to test animals against MI.

#### 5.3.5. 10-Gingerol

10-gingerol is a phenolic compound found in ginger, a popular spice for cooking. It has been reported to have a potential role in diminishing ROS levels in cells. Previous research showed that this phytocompound exhibits protective activity against MI in both in vitro and in vivo experiments. The underlying mechanism is activation of the JAK2/STAT3 signaling pathway and prevention of oxidative stress, leading to a reduction in apoptosis [138].

#### 5.3.6. Salvianolic Acid B

Salvianolic acid B (Sal B), derived from the herb *Salvia miltiorrhiza,* has numerous health benefits. It works against oxidative stress, inflammation, liver fibrosis, and cancer. Numerous in vitro and in vivo studies suggest that in myocardial damage models, Sal B inhibits ferroptosis by activating the Nrf2 signaling pathway, thereby offering protection against MI [139].

#### 5.3.7. Gallic Acid

Gallic acid, a phenolic substance present in many plants, such as gallnuts, sumac, witch hazel, tea leaves, oak bark, and grape seeds, has shown impressive outcomes in the treatment of ailments caused by oxidative stress. Several studies explored that the isoprenaline (ISO)-induced myocardial infarction (MI) in the rat model can possibly be improved by gallic acid [140,141]. The findings provide evidence that gallic acid reduces the oxidative damage induced by ISO, activates endogenous natural antioxidant enzymes, and guards the heart against MI.

The above-discussed seven phenolic compounds have been reported to show cardio-protection mostly via oxidative stress reduction, anti-inflammatory action, anti-apoptotic, and anti-ferroptotic functions. While all the compounds demonstrated promising cardioprotective activity in MI, almost all the evidence is backed by preclinical in vivo and in vitro data, except for curcumin, for which preliminary clinical evidence is available. Hence, these compounds should be assessed in clinical trials to find their therapeutic potential.

### 5.4. Alkaloids

#### 5.4.1. Nuciferine

Nuciferine, an aporphine alkaloid that is mostly found in *Nelumbo nucifera* leaves, has been linked to anti-cancer, anti-oxidant, anti-tumor, and insulin secretor actions. An investigation was carried out by HarishKumar and Selvaraj to assess the cardioprotective effects of nuciferine [142]. There, isoproterenol (ISO) was first used to cause MI in Wistar rats, and then nuciferine was administered orally as a pretreatment. In rats with MI, the pretreatment with nuciferine greatly raised the amount of endogenous antioxidants and reduced lipid peroxidation. In a different study on the acute myocardial infarction mouse model, Xie et al. found that the regulation of the PI3K/AKT pathway is responsible for the cardioprotective function of nuciferine [143]. In light of this, nuciferine may be considered for further investigation to assess its clinical applicability in MI patients.

#### 5.4.2. Berberine (Ber)

Doxorubicin (DOX)-induced MI damage has been acknowledged as a significant side effect of cancer chemotherapy. The goal of an experimental investigation by Y. Wang et al. [144] was to determine whether Ber, an isoquinoline alkaloid initially isolated from Chinese goldthread (*Coptis chinensis*), could offer defense against this problem. Berberine has a wide spectrum of biological roles, including antioxidant function. The study reveals that myocardial damage was prevented by pretreatment with Ber as it reduced the formation of ROS and MDA and raised SOD activity via triggering the Nrf2-mediated pathway. Despite demonstrating positive outcomes, Ber is also reported to have poor oral bioavailability [145].

#### 5.4.3. Liensinine

The *Nelumbo nucifera* Gaertn. seed embryo contains a naturally occurring bisbenzylisoquinoline alkaloid, liensinine (LSN), which has been shown to have several antioxidant and cardiovascular potentials. Research conducted by Shen et al. assessed the cardioprotective activities of LSN following MI, which demonstrated its novel cardioprotective function. This compound has significant potential to protect myocytes from oxidative stress-induced damage in MI by down-regulating the Wnt/β-catenin signaling pathway [146]. This finding supports the novel therapeutic role of LSN in ischemic heart diseases as well.

#### 5.4.4. Brucine

MI-related harms can be mitigated and significant advantages can be achieved by combining antioxidant and anti-inflammatory functions. In this context, the major bioactive alkaloid from *Strychnos nux-vomica* seeds, brucine, which has anti-inflammatory and antioxidant characteristics, could be quite effective in treating MI. An extensive study by Liu et al. showed that oral brucine preparation protected rat myocardium from ISO-induced myocardial damage. Brucine successfully reduced the extent of the infarct by boosting endogenous antioxidants, lowering the status of the marker enzymes TBARS and LOOH, and ameliorating histopathological damage. However, clinical application of brucine is limited because it has a narrow therapeutic index and produces systemic toxicity, most prominently, neurotoxicity [147,148,149].

### 5.5. Coumarins

#### 5.5.1. Fraxetin

Fraxetin is a coumarin derived from the *Fraxinus rhynchophylla* plant. Dual antioxidative action of fraxetin against metals and free radicals has been proven by Thuong et al. [150]. According to that study, fraxetin is a more effective free radical scavenger than other antioxidants such as esculetin and caffeic acid. Among numerous studies, Yin et al. looked into the cardioprotective abilities of fraxetin (Fx) in experimental rats with myocardial infarction [151]. The study showed that, besides improving antioxidant activity and decreasing free radicals, fraxetin also reduces cardiac tissue inflammation and damage, supporting its cardioprotective effects in rats following MI.

#### 5.5.2. Scopoletin

Scopoletin, a prominent phytoalexin present in many plant species, particularly those belonging to the Convolvulaceae, Ulmaceae, Solanaceae, Compositae, Oleaceae, Rutaceae, and Aceraceae families, has been revealed to have a strong capacity to scavenge free radicals. A pre-treatment with scopoletin, according to Rong et al. [152], significantly decreased the heart-to-body weight ratio, cardiac diagnostic markers, MDA, inflammatory indicators, and apoptotic markers, as well as elevated levels of antioxidant enzymes. As a result, histological features similar to inflammation and necrosis were reversed in the MI model.

#### 5.5.3. Auraptene

Auraptene is a citrus peel-derived component and has a variety of pharmacological properties that make it useful in treating MI. Sunagawa et al. found that auraptene drastically reduced MI-induced systolic dysfunction in MI-affected rats and improved posterior wall thickness [153]. Auraptene therapy was used to counteract MI-induced reductions in the expression of PPARα-dependent genes.

#### 5.5.4. Psoralidin (PSO)

Adriamycin (ADR) is an effective and widely used broad-spectrum anticancer medication. However, ADR’s cumulative and dose-dependent cardiotoxicity severely restricts its clinical use. A natural phenolic coumarin called psoralidin (PSO), which was reported to have reduced ADR-induced cardiotoxicity, is extracted from the seeds of the medicinal plant *Psoralea corylifolia* L. PSO demonstrates a variety of biological actions, including anti-inflammatory, anti-allergic, antibacterial, antidepressant, and antioxidant properties. According to Liang et al., PSO significantly decreased ADR-induced excessive ROS buildup in cardiomyocytes [154]. It also increased the levels of Nrf2, NQO1, and HO-1, suggesting that it may prevent oxidative stress and ultimately lessen the cardiotoxicity caused by ADR.

### 5.6. Saponins

#### 5.6.1. Ginsenoside Rg1

Ginsenoside Rg1 (Rg1) is a saponin of the protopanaxtriol type. It has the ability to provide protection to the mitochondria and ischemic myocardium. Rg1 has several functions, including the prevention of apoptosis and oxidative damage, as well as the stimulation of blood vessel and cardiomyocyte regeneration. An analysis by Yang, Jiang and Xing on the protective effect of Rg1 on myocardial ischemic injury in rats after AMI revealed that Rg1 may increase the rate of recovery of cardiomyocytes, lower the rate of cardiomyocyte apoptosis, lessen myocardial infarction area, elevate microvessel density in infract area, and preserve myocardial cells after AMI by means of antioxidant damage mechanisms [155]. It is therefore a potential therapeutic candidate for MI due to these attributes.

#### 5.6.2. Dioscin

Dioscin is a natural phytocompound present in numerous medicinal plants belonging to the *Dioscorea* and *Liliaceae* families. Several studies have shown that dioscin can lower oxidative stress and inflammation in rats with cardiac ischemia–reperfusion. Dioscin showed significant potential to prevent MI by controlling inflammation and oxidative stress that are mediated by BMP4/NOX1 [156].

#### 5.6.3. Notoginsenoside R1

In China, cardiovascular problems are frequently prevented and treated using *Panax notoginseng*, a well-known traditional Chinese medication. One of *P. notoginseng*’s main active ingredients, notoginsenoside R1 (NGR1), possesses anti-inflammatory, antioxidant, and anti-apoptotic activities [157]. According to Zhu and Wan, NGR1 produces superior therapeutic effects on MI [158]. The reporting of Xu et al. suggests that NGR1 controls the JAK2/STAT3 signaling pathway in hypoxia/reoxygenation (H/R)-treated H9C2 cells to enhance proliferation and prevent apoptosis [159]. NGR1 also slowed the in vivo course of MI.

### 5.7. Steroids

#### 5.7.1. β-Sitosterol

β-Sitosterol is a phytosterol isolated from *Nepeta deflersiana*. There is mounting evidence establishing β-sitosterol’s protective effects on cardiovascular illnesses. In a recent mechanistic investigation by Wong et al., it was discovered that β-sitosterol effectively increased cellular glutathione redox cycling, thereby reducing oxidative damage in rat cardiomyocytes [160]. Additionally, rat cardiac damage induced by isoproterenol was prevented by β-sitosterol. Moreover, β-sitosterol demonstrated protective effects against carbon tetrachloride-generated hepatotoxicity in rats via enhancing mitochondrial glutathione redox cycling. Another study by Lin et al. found that β-sitosterol provided protection against the damage that hypoxia/reoxygenation-induced cardiomyocyte injury and cardiac I/R injury caused to cells in culture. Also, the regulation of PPARc/NF-κB signaling after myocardial I/R injury may contribute to the β-sitosterol-mediated cardioprotective effects. However, developing oral formulations of β-Sitosterol could be problematic due to its poor oral bioavailability, restricted intestinal absorption, and rapid biliary excretion [161,162].

#### 5.7.2. Diosgenin

Diosgenin is a bioactive steroidal sapogenin molecule. It is found largely in *Rhizoma polgonati*, *Smilax china*, and *Trigonella foenum-graecum,* along with other plants. Liu and his team evaluated the cardioprotective effects of the diosgenin molecule on MI mouse models. The study revealed that diosgenin minimizes oxidative stress in myocardial infarction-damaged cardiac tissue of rats. Diosgenin also has cholesterol-lowering traits, which may assist in preventing and controlling cardiovascular disease. Another study on the MI model of Wistar rats exhibited that diosgenin, along with exercise, shows cardio-protective activity because it has antioxidant and free radical scavenging potential. Nevertheless, diosgenin has low aqueous solubility and poor bioavailability, which may limit its therapeutic translation [163,164,165,166,167].

### 5.8. Phenylpropanoids

#### 5.8.1. Sinapic Acid

Sinapic acid (SA) is a widely distributed, orally accessible phytochemical with anti-inflammatory and peroxynitrite-scavenging properties. It is present in various foods, including cereals, citrus, berry fruits, vegetables, cereal grains, and oilseed crops. SA was observed to show an altered lipid profile, reduced myocardial infarct size, and improved lysosomal membrane damage in ISO exposed rats. Research was conducted to divulge the protective function of SA on cardiac mitochondrial damage in rats with ISO-induced myocardial infarction [168]. It was found that pretreatment and cotreatment with SA elevated the levels of antioxidants and protected heart mitochondria in ISO-treated rats by reducing oxidative stress.

#### 5.8.2. Ferulic Acid

Ferulic acid (FA), a polyphenol generated from plants, is widely present in cereals, vegetables, and fruits. FA is well known for its anti-inflammatory properties. According to Pandi et al. [169], FA’s cardio-protective effects mainly involve modulating cellular antioxidants, ROS-mediated oxidative stress, apoptosis, inflammation, and autophagy. FA primarily modulates the PI3K/Akt-dependent Nrf2 signaling pathway.

### 5.9. Organosulfur Compounds

#### 5.9.1. Allicin

Allicin, a very effective natural antimicrobial compound found in garlic, prevents the growth of numerous pathogens [170]. Gao et al. demonstrated that allicin could alleviate myocardial infarction in I/R by exerting a cardioprotective effect via promoting the SHP2 axis to inhibit p-PERK-mediated oxidative stress [171]. Allicin was utilized to treat rats using a myocardial infarction rat model in order to study the effects of allicin on myocardial infarction [172]. It demonstrated how allicin works with oxidative stress markers, apoptosis-related proteins, and the JNK signaling cascade to minimize oxidative stress damage and cardiomyocyte death in a rat model of myocardial infarction. Hence, the therapeutic impact of allicin on myocardial infarction should be further investigated using a large sample size of clinical patients. Chemically, allicin belongs to organo-sulfur compounds. In addition to their desirable bioactivity, these sulfur compounds may also exhibit toxicological effects. The demonstration of their toxicological effects relies on the route and amount of exposure, as well as on their chemical forms [173]. Allicin is also reported for its instability, rapid degradation and metabolism [130,174].

#### 5.9.2. α-Lipoic Acid

α-lipoic acid (ALA) is a powerful antioxidant that occurs in nature and has a wide range of antioxidant potential that may increase its effectiveness in disease states where oxidative stress plays a vital role in cardiovascular pathophysiology. In a mouse model of AMI, the cardioprotective potential of ALA was investigated by Yang et al. [175]. The study showed that daily administration of ALA not only decreased oxidative stress during both the acute phase immediately following an AMI and the chronic remodeling phase that followed, but also protected the heart from future attacks and helped maintain cardiac structure and function throughout the progression of LVR post-AMI. However, α-lipoic acid is also associated with extensive first-pass hepatic metabolism and has a short plasma half-life [176]. Despite having these shortcomings, ALA underwent a preliminary clinical trial and showed positive outcomes, which is further discussed in detail in Section 8.

### 5.10. Carotenoids

#### 5.10.1. Lycopene

Experiments by Asgary et al. and Guo, Huang and Li provided a theoretical basis for the antioxidant and cardioprotective effects of lycopene [80,177]. Guo, Huang and Li demonstrated that lycopene can effectively neutralize free radicals and stimulate the transcription of downstream antioxidant enzymes via activating the Nrf2/HO-1 pathway. It is noteworthy to mention that the Nrf2/HO-1 pathway is considered a key antioxidant defense system of the body. The participation of this pathway in the antioxidant mechanism of many compounds shows that this pathway might represent a common convergent mechanism, which may contribute to their cardio-protective activity. In another clinical trial, Asgary et al. also observed the cardio-protective activity of lycopene in patients undergoing PCI. The preliminary clinical trial of lycopene is discussed in detail in Section 8.

#### 5.10.2. Lutein

Lutein (LU) is a naturally occurring plant-derived oxygenated carotenoid. It is found in a variety of fruits and vegetables, particularly in spinach and egg yolks. LU has been reported to show numerous pharmacological properties, such as anti-inflammatory, antioxidant, and anti-apoptotic actions. A study designed by Abdelmonem et al. investigated the possible preventative role of LU against ISO-induced MI. The study concluded that LU may lessen the severity of the condition in rats by improving endogenous antioxidant defense and regulating the MIAT/miR200a/Nrf2 pathway. Although LU exhibited positive outcomes in in vivo studies, its translational applicability may be challenged because it has poor oral bioavailability [178,179,180].

### 5.11. Other Classes

#### 5.11.1. Shikimic Acid

Shikimic acid (SA), a plant-based Shikimate-derived compound, is one of the major intermediate products formed in the shikimate pathway in a number of plants [181]. It exhibits notable pharmacological effects, including antioxidant [182], anti-inflammatory [183], anti-platelet, and antithrombotic activities [184]. SA has been shown to reduce MDA levels and enhance SOD activity, thereby minimizing oxidative stress and cellular damage [185]. Moreover, it reduces pro-inflammatory cytokines, such as TNF-α and IL-1β, which contribute to ventricular remodeling, inflammation, and cardiomyocyte apoptosis after myocardial infarction [183].

#### 5.11.2. Emodin

It has been demonstrated that the principal active ingredient in traditional Chinese medicine, rhubarb, an anthraquinone derivative, emodin, possesses anticancer, antibacterial, immunomodulatory, and antioxidant properties. To generate fresh concepts for the clinical management of post-MI heart failure (MI HF), the effect of emodin on myocardial cells was investigated in an experimental rat model by Liu and Ning [186]. According to the research, emodin has the potential to be a therapeutic option because it can considerably enhance energy metabolism, lower the pace at which myocardial tissues undergo apoptosis, and improve cardiac function in post-MI HF rats.

#### 5.11.3. Swertiamarin

Swertiamarin is a naturally occurring plant glycoside. Chemically, it belongs to iridoids. It is found abundantly in *Enicostemma littorale Blume* (*E. littorale*) along with a variety of other plant species. Swertiamarin is reported for its anti-diabetic, antinociceptive, antilipidemic, hepatoprotective, anti-obesity, anti-malarial, anti-leprosy, antioxidant, and anti-inflammatory activities [187]. The cardioprotective effect of swertiamarin was investigated by Wang et al., who demonstrated that pre-treatment with swertiamarin reduced oxidative stress while increasing levels of cellular antioxidants in MI rat models [188]. The underlying mechanism could be attributed to its ability to lower ROS by scavenging ROS radicals and by simultaneously activating the genes that encode antioxidant/antioxidant enzymes. The study also found decreased levels of the pro-inflammatory cytokines TNF-α and IL-6 and a significant protection against histological changes in rats with ISO-induced MI.

**Table 2:** Reported natural bioactive compounds along with their mechanistic insights into cardioprotection in MI, physicochemical properties, typical experimental dose and pharmacokinetic limitations.

Compound	Source	Chemical Class	Physicochemical Properties		Assay	Model	Typical Experimental Dose	Effect & Mechanism	Pharmacokinetic Limitations	Application	Reference
			Solubility	Lipophilicity (XLogP3-AA)							
Scopoletin	Convovulaceae (*convolvulus tricolor*), Ulmaceae, Solanaceae	Coumarins	Limited-to-moderate water-soluble	1.5	In vivo	Albino Sprague-Dawley male rats	25 and 50 mg/kg	Prevents oxidative stress by reducing MDA level and by increasing the levels of antioxidant enzymes (SOD and CAT)	Low bioavailability, rapid absorption, and extensive metabolism	MI	[152,189]
Shikimic acid	*Illicium verum*, *Liquidambar styraciflua*, *Pinus sylvestris*, *Malus pumila*	Shikimate-derived compounds	Highly water-soluble	−1.7	In vitro, in vivo	Sprague-Dawley rats	10, 30, and 50 mg/kg	Decreases MDA level and increases SOD activity, thereby decreasing the cellular damage caused by free radicals	Low oral bioavailability and rapid systemic clearance	MI	[185,190]
Nerolidol	Lavender, lemon grass, and ginger	Terpenoids	Slightly water-soluble	4.6	In vivo	Male Wistar normotensive rats	50 and 100 mg/kg	Increases SOD activity and reduces lipid peroxidation and carbonyl levels	Poor water solubility, rapid degradation and low bioavailability	MI	[109,114]
Biochanin A	Red clover, soy, alfalfa sprouts, peanuts, and chickpeas	Flavonoids	Poor water-soluble	2.9	In vivo	Male Wistar rats	10 mg/kg	(i) Protects from oxidative stress by enhancing the levels of GPx, GST, GSH, and GRD. (ii) Increases activities of enzymatic antioxidants, such as (SOD) and (CAT) & reduces MDA levels	Poor aqueous solubility, extremely low oral bioavailability, and extensive first-pass metabolism	MI	[102,191]
Diosmetin	Lemon peel and Citrus Fruit (*Olea europaea* L.)	Flavonoids	Poor water-soluble	2.8	In vivo, In vitro	Rat model, H9c2 cell line	1 and 3 mg/kg	(i) Protects against oxidative stress by inhibiting the generation of MDA and restoring the antioxidant enzymes (SOD, GPX, CAT) (ii) Activates ꞵ 1-adrenergic receptors (iii) Reduces apoptosis of myocardium via the autophagy induction by activating AMPK	Extremely low water solubility, poor gastrointestinal absorption, and extensive first-pass metabolism	MI	[97,98,192]
10-gingerol	Ginger	Phenolics	Slightly water-soluble	3.6	In vivo	Sprague-Dawley (SD) rats	20 and 40 mg/kg	Minimizes oxidative stress by reducing ROS & MDA & increasing SOD, CAT, and GSH levels; activates JAK2/STAT3 pathway	Low bioavailability, rapid first-pass metabolism, and a short elimination half-life	MI	[138,193]
Quercetin	Apple, onion, tea	Flavonoids	Very low water-soluble	1.5	Clinical trial	Human	500 mg	May increase the content of nitric oxide & metabolism of leukotriene and thereby decrease oxidative stress & inflammation	Low oral bioavailability, poor water solubility, gastrointestinal degradation, and rapid phase II metabolism	ST-elevated MI	[85,86]
Lycopene	Tomato	Carotenoids	Practically insoluble in water	10.0	In vitro, clinical trial	HMEC-1 cell, ECV-304 cell, human	30 mg/individual, 15 mg/individual	(i) Minimizes oxidative stress by declining ROS & MDA levels, restores GSH, GCLC, GCLM expression & activates the SIRT1/Nrf2/HO-1 pathway (ii) Cardioprotective effect by reducing CKMB	Poor aqueous solubility, lipophilicity and saturable intestinal absorption	PCI	[80,177,194]
Berberine	Chinese goldthread (*Coptis chinensis*)	Alkaloids	Low water-soluble	1.5–2.0	In vivo	Wild-type male SD rats	60 mg/kg	Inhibits oxidative stress and mitochondrial injury by up-regulating the expression of Nrf2, HO1, TFAM, as well as decreasing MDA & increasing SOD levels	Poor oral absorption and low bioavailability	Myocardial injury	[144,145]
Ellagic acid	Pomegranates, blackberries, raspberries, strawberries, cranberries, walnuts, pecans, wolfberry	Phenolics	Very low water-soluble	2.0	In vivo	Female Wistar rats	30 mg/kg	Increases SOD activity and reduces ROS & CaMKII phosphorylation	Low solubility, limited intestinal absorption and large interindividual variability in bioavailability	MI-induced LVDD	[132,195]
Thymoquinone	*Nigella Sativa*	Terpenoids	Poor water-soluble	2.2	In vivo	Male albino rats	20 mg/kg	Decreases MDA & ROS, & significantly elevates GSH, GPx, SOD, CAT, as well as preserves the cardiac mtDNA content	Poor oral bioavailability and low solubility	MI	[126,196]
Epigallocatechin gallate	Green tea (*Camellia sinensis*)	Flavonoids	Moderate water-soluble	1.0	In vitro, in vivo	H9C2 cardiomyocytes, male C57BL/6 mice	50 mg/kg	(i) MDA levels & the expression of Bax & p-PI3K/PI3K, p-Akt/Akt are significantly decreased, and the activity of SOD & expression of Bcl-2 are significantly increased (ii) Antihypertensive effect by reducing oxidative stress through down-regulation of vascular NADPH oxidase activity	Low bioavailability, extreme chemical instability, and rapid bodily clearance	Myocardial Ischemia–Reperfusion Injury (MIRI)	[89,90,91]
Lupeol	Olive Oil, Strawberry	Terpenoids	Practically insoluble in water	7.0	In vivo	SD rats	50 mg/kg	Suppresses the ROS & MDA expression & increases the level of SOD, GSH, GPx, as well as enhances the expression of Nrf2/HO-1	High lipophilicity, poor water solubility and low bioavailability	MIRI	[116,197]
S-limonene	Lemons and Oranges peel	Terpenoids	Practically insoluble in water	4.5	In vivo	Wistar rats	1 mg/kg	Inhibits increased Ca^2+^ and attenuates oxidative stress via CaMKII, as well as causes the restoration of SOD and GPx activity	Low metabolic and plasma stabilities, low bioavailability and tissue distribution/accumulation	MI	[81,105]
Ferulic acid	Grain bran, Whole grain foods, Citrus fruits, Banana, Coffee, Cabbage, Celery and Carrots	Phenylpropanoids	Moderate water-soluble	1.5	In vitro, in vivo	H9c2 cell, C5/BL/6 J mice	10, 20 and 40 mg/kg	Reduces oxidative stress by alleviating MDA, Restores SOD, CAT, GPx, GST & GSH levels, as well as minimizes apoptosis and activates Nrf2 signaling pathway	Low absolute bioavailability, rapid liver metabolism and poor water solubility	Arrhythmia, MI, Myocardial Hypertrophy	[169,198]
Kaempferol	*Amaranthus viridis*	Flavonoids	Very low water-soluble	1.9	In vivo	Male Wistar rats	50 mg/kg	Attenuates oxidative stress by elevating GSH, and decreasing lipid peroxidation, along with suppressing ROS & NOx, in addition to restoring the activity of SOD, CAT, GPx, GR, GST, and up-regulating the Nrf2/HO1 pathway	Low to moderate absorption, low bioavailability and extensive first-pass metabolism	MI & Post-MI Damage	[101,199]
Icariin	*Epimedii Herba*	Flavonoids	Very low water-soluble	2.0	In silico	—	—	Potential targets are EGFR, AKT1, TP53, JUN, ESR1, PTGS2, TNF, RELA, HSP90AA1, BCL2L1 & protective effect is associated with inhibited oxidative stress	Low water solubility, low membrane permeability, slow dissolution rate and poor oral bioavailability	MI	[94]
Liensinine	*Nelumbo nucifera Gaertn*	Alkaloids	Very low water-soluble	3.8	In vitro, in vivo	Human AC16, rat H9c2 cells, male C57BL/6 mice	10 mg/kg	Protects against ischemic and oxidative stress-induced DNA damage via inhibiting Wnt/β-catenin signaling pathway activation	Poor absorption and extremely low oral bioavailability	MI-induced Ischemic Injury	[146,200]
Taraxerol	Asteraceae family (*Taraxacum officinale*)	Terpenoids	Practically insoluble in water	7.0	In vivo	Male SD rats	20 and 40 mg/kg	Decreases MDA levels & increases the activity of SOD and GPx	Poor aqueous solubility and low bioavailability	MI	[123,201]
Dioscin	*Dioscorea nipponica Makino*	Saponins	Low-to-moderate water-soluble	1.2	In vitro, in vivo	HL-1 cells, C57BL/6 mice	20, 40 and 80 mg/kg	Increases the viability of HL-1 cells and inhibits ROS level along with decreasing LDH, CK-MB, cTnI, MDA & increasing SOD by down-regulating BMP4/NOX1-mediated oxidative stress	Poor aqueous solubility and unstable in water	MI Injury	[156,202]
Salvianolic acid B	*Salvia miltiorrhiza*	Phenolics	Moderate water-soluble	0.5	In vivo	SPF-grade male rats	25 and 50 mg/kg	Inhibits MI-induced ferroptosis by alleviating iron overload and oxidative stress through activating the Nrf2 signaling pathway	Very hydrophilic, poor stability in water and low absolute bioavailability	MI	[139,202]
Hinokitiol (β-thujaplicin)	Cupressaceae (*Chamaecyparis obtusa*)	Terpenoids	Low-to-moderate water-soluble	2.0	In vitro	Human AC16 cells	NA	Protects cardio-myocytes by increasing p21 expression through GSK3β/p21 signaling	Poor aqueous solubility, rapid degradation, and low intestinal absorption	MIRI	[125,203]
Saprirearine	*Salvia prionitis*	Terpenoids	Low water-soluble	2.5	In vitro	Rat H9c2	—	Oxidative state is reversed by decreasing ROS & MDA & increasing the activity of SOD and CAT. It also activates Nrf2 pathway	—	MI	[122]
Auraptene	*Citrus hassaku*	Coumarins	Low water-soluble	4.5	In vitro, in vivo	Rat neonatal cardiomyocytes, SD rats	5 and 50 mg/kg	Represses MI-induced cardiac hypertrophy & left ventricular systolic dysfunction by activating PPARα	Low oral bioavailability, extensive first-pass metabolism, high lipophilicity, and rapid enzymatic clearance	MI	[153,204,205]
Notoginsenoside R1	*Panax notoginseng*	Saponins	Low intrinsic water solubility	1.5	In vitro, in vivo	Rat H9C2 cell, male SD rats	20 and 40 mg/kg	Regulates the proliferation and apoptosis of H9C2 cells & attenuates MI by activating the JAK/STAT3 signaling pathway.	Low oral bioavailability, poor membrane permeability, rapid first-pass metabolism by gut microbiota and biphasic elimination profile	MI	[159,206]
Salidroside	*Rhodiola rosea* L.	Phenolics	Highly water-soluble	−0.5	In vivo	Male wild-type SD rats	50 mg/kg	Increases SOD and CAT activities and decreases MDA and LDH content, along with significantly promoting p-PI3k, p-AKT, Nrf2 and HO-1 expression	Rapid plasma clearance and low oral bioavailability	MI	[136,207]
Psoralidin	*Psoralea corylifolia* L.	Coumarins	Low water-soluble	3.8	In vitro, in vivo	HL-1 cells, male BALB/c mice	12.5, 25, and 50 mg/kg	Upregulates expression of Bcl2/Bax, Nrf2, NQO1, HO-1, NRF1, TFAM, UCP2, and inhibits oxidative stress by upregulating the expression of Nrf2,HO-1,NQO1	Highly polar and easily metabolized	Cardiotoxicity (MI, HF)	[154,208]
Calycosin	*Radix Astragali*	Flavonoids	Low water-soluble	2.8	In vitro, in vivo	Neonatal cardiomyocytes. C57BL/6 male mice	10 and 20 mg/kg	Inhibits cell apoptosis and oxidative stress by reducing ROS,4-HNE & MDA levels via activating ALDH2	Poor circulating levels, extensive first-pass metabolism and high tissue sequestration	MI	[96,209]
Emodin	Rhubarb	Quinones	Low water-soluble	2.7	In vivo	Male SD rats	20, 40, and 60 mg/kg	Decreases levels of cTnI and PGC-1 & the expressions of complex I and p-ERK in myocardial tissues	Extremely low bioavailability, poor absorption and extensive phase II metabolism	Post-MI HF	[186,210]
Nuciferine	*Nelumbo nucifera*	Alkaloids	Low water-soluble	3.3	In vivo, in silico	Male Wistar rats	10 and 20 mg/kg	Mitigates oxidative stress by decreasing ROS & MDA levels along with increasing SOD, CAT, GSH, and enhances the up-regulation of Bcl-2, in addition to the down-regulation of Bax, Cas-9 & Cas-3	low oral bioavailability, poor absorption, rapid metabolism, quick elimination, and poor water solubility	MI	[142,211]
Gallic acid	Gallnuts, Sumac, Witch hazel, Tea leaves, Oak bark, and Grape seeds	Phenolics	Highly water-soluble	0.7	In vivo	Male albino rats	15 mg/kg; 25 and 50 mg/kg	Attenuates the oxidative stress by restoring the activity of SOD, GSH & decreases MDA level.	Low absorption, low bioavailability, high metabolism, and clearance	MI	[140,141,212]
Diosgenin	*Rhizoma polgonati*, *Smilax china*, *Trigonella foenum-graecum*	Steroids	Practically insoluble in water	5.0–5.5	In vivo	Mice, male albino Wistar rats	10 mg/kg	Minimizes oxidative stress by increasing activity of SOD, CAT, GSH, GPx, GST & reducing ROS formation	Low aqueous solubility, poor bioavailability, and rapid biotransformation	MI	[163,164,165,166,167]
Puerarin	*Radix puerariae*	Flavonoids	Moderate water-soluble	0.6	In vivo	Male SD rats	120 mg/kg	Activates the PI3K/Akt signaling pathway	Low oral bioavailability, poor solubility and low membrane permeability	AMI	[99,213]
Bakuchiol	*Psoralea Corylifolia*	Phenolics	Very low water-soluble	4.8	In vivo	Wild-type C57/BL 6N mice	60 mg/kg	Produces cardioprotective effects by inhibiting the gene expression of ERK2 and TGF-β1	Poor bioavailability, limited water solubility and extensive first-pass metabolism	MI	[121,214]
β-Sitosterol	*Nepeta deflersiana*	Steroids	Practically insoluble in water	8.0	In vitro, in vivo	H9c2 cells, SD rats	NA	Reduces oxidative stress and promotes mitochondrial function by inhibiting cell apoptosis and ROS production, and increasing MMP	Poor oral bioavailability, restricted intestinal absorption and rapid biliary excretion	MIRI	[161,162]
Allicin	Garlic	Organosulfur compounds	Moderate water-soluble	1.0	In vivo	Male SD rats	1.2, 1.8 and 3.6 mg/kg	Increases SOD, CAT, GSH-Px activities and decreases MDA levels, along with significantly regulating the JNK signaling pathway	Rapid degradation and metabolism	MI	[130,172]
Alpha-Lipoic Acid	Broccoli	Organosulfur compounds	Moderate water-soluble	2.2	In vivo	C57Bl/6 mice	15 and 75 mg/kg	Reduces oxidative stress by decreasing ROS & MDA & plasma nitrate/nitrite & Myeloperoxidase	Poor gastric stability, low aqueous solubility, and extensive first-pass hepatic metabolism	AMI	[175,176]
Taxifolin	Olive Oil, Grapes, Onions	Flavonoids	Low water-soluble	1.5	In vivo	Swiss albino mice	25 and 50 mg/kg	Attenuates cardiac injury by CK-MB, cTnI, LDH, and protects against oxidative stress via reduction in MDA, protein carbonyl, and NO contents, and increases SOD, CAT, GSH, as well as activates cardiac Nrf2/HO-1 signaling pathway	Poor aqueous solubility, nonlinear pharmacokinetics, and extensive gastrointestinal and hepatic metabolism	MI Other CVDs	[103,215]
Nootkatone	Grapefruit	Terpenoids	Low water-soluble	4.3	In vivo	Male Wistar albino rats	10 mg/kg	Protects myocardium by inhibiting the release of CK, LDH, & troponin-T into the serum, minimizes oxidative stress by increasing the activities of SOD, CAT, GSH, vitamin C, vitamin E via modulation of PI3K/Akt/Nrf2 signaling pathway	Low aqueous solubility and poor environmental stability	MI	[117,216]
Formononetin	*Trifolium pratense* (red clover)	Flavonoids	Low water-soluble	2.9	In vitro, in vivo	NCMs, male SD rats	10 and 30 mg/kg	Mitigates the elevation of ROS & inhibits the activation of NLRP3 inflammasome via the suppression of ROS-TXNIP-NLRP3 pathway	Low water solubility and poor oral bioavailability	MIRI	[88,217]
Curcumin	Turmeric (*Curcuma longa*)	Phenolics	Practically insoluble in water	3.3	In vitro, in vivo	Wistar rats’ cardiac fibroblasts, wild-type male mice	100 mg/kg	Alleviates cardiac fibrosis by regulating collagen deposition, ECM degradation, and CFs’ proliferation and migration, as well as producing antioxidative stress effects by attenuating the down-regulation of SIRT1	Extremely low oral bioavailability, poor water solubility, limited gastrointestinal absorption, and rapid metabolism	MI-induced cardiac fibrosis	[128,218]
Baicalein	*Scutellaria baicalensis*, *Scutellaria lateriflora*	Flavonoids	Low water-soluble	2.3	In vivo, in vitro	Male Wistar albino rats	50 mg/kg and 100 mg/kg	(i) Exerts significant antioxidant activity by declining nitric oxide and lipid peroxidation (MDA) and enhancing SOD, catalase and GSH activity (ii) Suppresses ferroptosis in cardiomyocytes by reducing ROS and MDA	Highly poor water solubility, low dissolution and absorption	MIRI MI	[92,219,220]
Luteolin	Carrots, Olive Oil, Peppers, Rosemary	Flavonoids	Low water-soluble	2.4	In vitro, in vivo	H9c2 cell, male SD rats	10, 40, and 70 mg/kg	(i) Increases T-SOD activity, and decreases LDH, CK, MDA levels, along with decreasing the ROS generation and modulation of MAPK pathway (ii) Suppresses ferroptosis in cardiomyocytes by reducing ROS and MDA	Low water solubility and rapid, active efflux by P-glycoprotein in the gastrointestinal tract	MIRI MI	[93,219,221]
Brucine	*Strychnos nux-vomica* seeds	Alkaloids	Low water-soluble	2.3	In vivo	Male Wistar rats	50 mg/kg	Increases enzymatic antioxidant activities such as SOD, CAT, GPx, GSH, GSSG, GSH/GSSG ratio, and elevates the Na+/K+-ATPase activity	Rapid clearance, neurotoxicity, and narrow therapeutic window	MI	[147,148,149]
Sinapic acid	Citrus and Berry fruits, Vegetables, Cereals and Oilseed	Phenylpropanoids	Moderate water-soluble	1.2	In vivo	Male Albino Wistar rats	3, 6, and 12 mg/kg	Prevents oxidative stress by reducing the level of TBARS & improving SOD, GPx, and GSH levels, as well as protects the heart mitochondria for its anti-lipid peroxidation effect	Poor aqueous solubility, low in vitro dissolution rate, and rapid metabolism	MI	[168,222]
Tanshinone IIA	*Salvia miltiorrhiza* Bunge	Terpenoids	Low water-soluble	4.3	In vivo	Male SD rats	1.5 mg/kg	Attenuates oxidative stress by decreasing MDA, superoxide anions and Nox activity levels & by increasing SOD activity	Low oral bioavailability, poor water solubility and limited membrane permeability	MI	[108,221]
Rosmarinic acid	*Rosmarinus officinalis*	Phenolics	Moderate water-soluble	1.0	In vivo	Rats	25 and 50 mg/kg	Exerts potent cardio-protective effects by increasing BDNF, IL-10, GSH, and SOD activity	Extremely low absolute bioavailability, poor intestinal membrane permeability and rapid metabolism	Depression-induced MI	[137,223]
Swertiamarin	*Enicostemma littorale* Blume	Iridoids	Highly water-soluble	−1.0	In vivo	Albino male Wistar rats	20 and 40 mg/kg	Alleviates oxidative stress by reducing MDA, PC levels, and raising GSH, SOD, CAT, GPx, GST, GR, and TAC levels	Low oral bioavailability, poor permeability and first-pass effect	MI	[188,224]
Fraxetin	*Fraxinus rhynchophylla*	Coumarins	Moderate water-soluble	1.3	In vivo	Male Wistar rats	50 mg/kg	Reduces oxidative stress by preventing the rise in MDA levels & elevating the levels of SOD, GSH, CAT, and GPx, along with blocking lipid peroxidation & increasing Na+/K+ATPase levels	Low oral bioavailability and rapid first-pass metabolism	MI	[151,225]
Ginsenosides Rg1	*Panax ginseng*	Saponins	Moderate-to-high water-soluble	1.0–2.0	In vivo	Wistar rats	10, 20 and 40 mg/L	Scavenges oxygen free radicals by SOD and GSH-Px contents	Low oral bioavailability, poor membrane permeability, decomposition in the stomach, intestinal metabolism, and hepatic elimination	MI	[155,179]
Lutein	Spinach and Egg Yolks	Carotenoids	Practically insoluble in water	8.0	In vivo	Male Wistar rats	20 mg/kg	Reduces ROS by Nrf2 activation, leading to the down-regulation of TXINP expression that increases thioredoxin and GSH content	Low aqueous solubility and low oral bioavailability	MI	[178,179]
α-bisabolol	*Chamomilla recutita* L., *Salvia runcinata*, *Plinia cerrocampanens*, *Eremanthus erythropappus*	Terpenoids	Low water-soluble	4.0	In vivo	Male albino Wistar rats	25 mg/kg	Exerts free radical scavenging effect by reducing the concentrations of lipid peroxidation products & restoring the concentrations of GSH and vitamin C	Poor aqueous solubility and low bioavailability	MI	[120,226]

Note: information related to the physicochemical properties of the compounds included in Table 2 was accessed from PubChem database (https://pubchem.ncbi.nlm.nih.gov, accessed on 13 July 2026).

## 6. Translational Limitations and Challenges of Natural Antioxidant Compounds

Although plant-derived antioxidant compounds have demonstrated potential cardio-protective activities against MI, their therapeutic efficacy basically relies on in vivo bioavailability and other pharmacokinetic factors in addition to in vitro antioxidant potential [227,228,229]. Usually, most plant-derived antioxidant compounds, including polyphenols and flavonoids, are known for their poor bioavailability problems due to low aqueous solubility, low intestinal absorption, extensive pre-systematic metabolism, chemical instability, and rapid elimination [230]. Thus, their formulation and delivery become challenging, and this necessitates the development of advanced drug delivery systems such as nanoparticles, liposomes, phospholipid complexes, and micelles [231]. For instance, a well-known flavonoid quercetin has markedly enhanced bioavailability when prepared in nanoformulations [86]. While choosing natural antioxidants as therapeutics, another critical issue to consider is that they might lack target specificity. Even though multiple molecular targeting may play a role in rendering these compounds efficacious, this may also lead to unwanted biological activities. It occurs because numerous antioxidants tend to suppress not only pathological ROS but also ROS associated with normal physiological processes [232]. Variation in the extraction and purification process of plant-derived antioxidant compounds may also impact the reproducibility of their biological effects [233]. Moreover, despite the report of many natural antioxidants being effective against MI in pre-clinical trials, only a few have undergone clinical trials. Hence, clinical data for most of the compounds are not available until now. Even the compounds undergoing clinical trials need further testing on a large scale to generate sufficient and verified data regarding safety, efficacy, bio-distribution, pharmacokinetics, optimal dosage, and clinical application. It is important to mention that sometimes inconsistencies between preclinical and clinical outcomes are observed, and this is why natural antioxidant compounds having good preclinical results must also be tested from a clinical point of view. Potential toxicity of antioxidants and their metabolites when consumed at high concentrations is another concern. Therefore, a precise risk/benefit assessment should be carried out prior to translating antioxidant supplementation into therapeutics [234].

## 7. Reported SAR of Natural Antioxidant Compounds Investigated for MI

### 7.1. SAR of Quercetin

**Figure 4:**
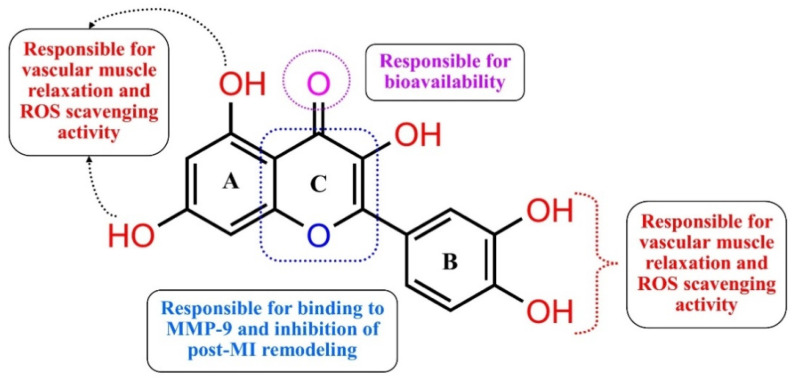
SAR of quercetin.

Quercetin is a phenolic compound, specifically a flavonol. It contains a specialized polyphenolic structure composed of two benzene rings linked by a pyrone ring. It contains five hydroxyl groups (-OH) responsible for donating hydrogen atoms, which help in the neutralization of reactive oxygen species (ROS), resulting in antioxidant defense in MI (Figure 4). In myocytes, -OH groups in ring A and B function as free radical scavengers and facilitate the relaxation of vascular tissues. The C pyrone ring containing C2=C3 and 4-carbonyl group is useful for binding to matrix metalloproteinase 9 (MMP-9), leading to enhancing recovery of myocardial tissue and lessening post-MI remodeling. Bioavailability is directly impacted by O-glycosylation substitutions. Derivatives, such as quercetin-3-O-glucoside, exhibit tissue-specific targeting and altered bioavailability [235,236].

### 7.2. SAR of Resveratrol

**Figure 5:**
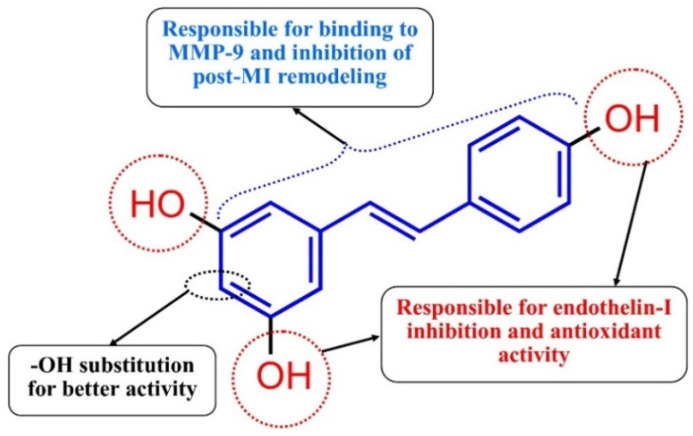
SAR of resveratrol.

A stilbenoid polyphenol, resveratrol, is made up of two benzene rings joined by an ethylene (1,2-diphenylethylene) backbone. Three hydroxyl groups (-OH) are joined to the benzene rings at particular locations to form the core structure, which is trans-3,5,4′-trihydroxystilbene. Stilbene backbone, along with its arrangement, might exert cardioprotective activity in MI (Figure 5). Modification of this core leads to a lack of inhibition of endothelin-1, which enhances myocardial necrosis and arrhythmogenesis, thereby worsening the MI. Apart from that, the number and position of –OH groups are directly related to endothelin-1 inhibition activity. Modification is possible only in the case of –OH groups. For example, 2 –OH groups in the 4 and 4′ position form 4,4′-dihydroxy-trans-stilbene, which is reported to show more potent cardioprotective activity than resveratrol. The antioxidant activity of this compound is also due to the presence of multiple –OH groups [237].

### 7.3. SAR of Ellagic Acid

**Figure 6:**
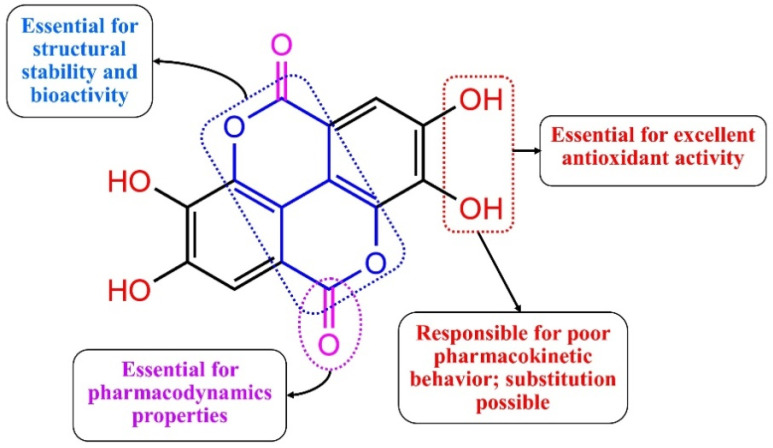
SAR of ellagic acid.

Ellagic acid, a natural polyphenolic compound, is reported to show effectiveness in MI treatment due to its antioxidant and anti-inflammatory activities. The presence of multiple hydroxyl groups (-OH) at positions 3, 3′, 4, and 4′ enhances its radical scavenging capacity, reducing oxidative stress and lipid peroxidation in myocytes. Oral bioavailability of this compound is affected due to the polar –OH moieties. In addition, the polar moieties are also responsible for extensive phase-II metabolism and rapid clearance. Cellular signaling for inflammation, as well as optimal interaction with molecular targets, is dependent on conjugated (C=O) structure (Figure 6). Apart from that, the presence of two lactone rings facilitates structural stability as well as bonding to target sites [238,239,240].

### 7.4. SAR of Biochanin A

**Figure 7:**
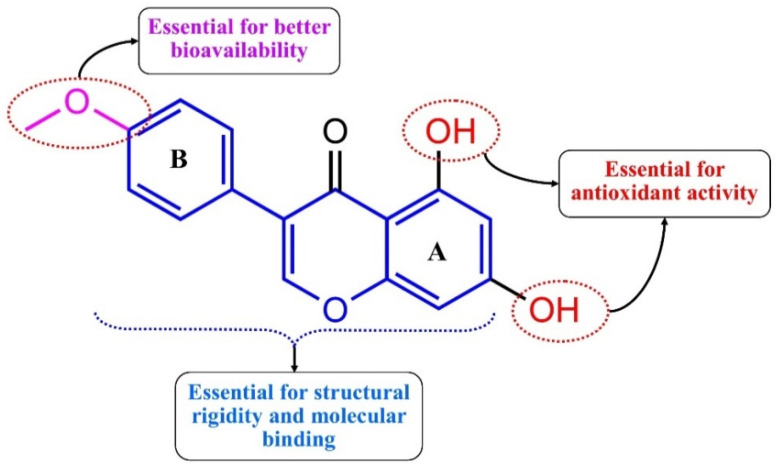
SAR of Biochanin A.

Biochanin A is an O-methylated isoflavone containing a core isoflavone scaffold attached with a number of substituents. Isoflavone scaffold, also known as 3-phenylchromen-4-one, is thought to maintain structural rigidity and affinity to the target site for vasodilation during MI. In ring A, the 5,7-dihydroxy groups (-OH) may mediate antioxidant activity, leading to the prevention of oxidative damage of myocytes during MI. In B ring, the 4′-methoxy group (C-O-C) facilitates oral absorption due to its lipophilic nature, thus increasing oral bioavailability (Figure 7). Apart from that, it facilitates the blocking of cytokine expression and prevents inflammation [241,242].

### 7.5. SAR of 10-Gingerol

**Figure 8:**
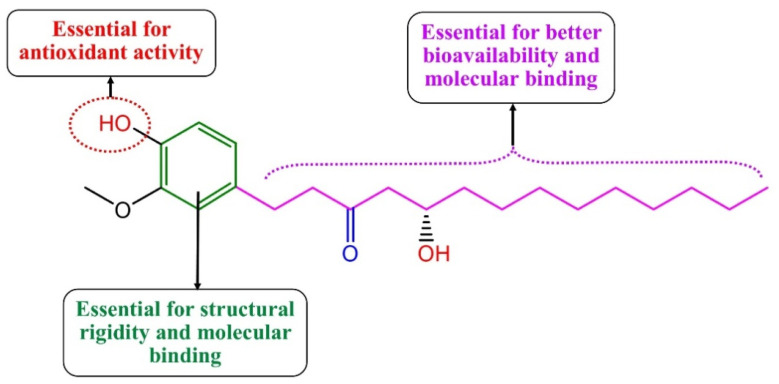
SAR of 10-gingerol.

10-Gingerol, a phenolic compound, is a beta-hydroxy ketone, specifically a 5-hydroxydecan-3-one substituted by a 4-hydroxy-3-methoxyphenyl moiety at position 1. The presence of an aromatic ring in the compound might be essential for the stabilization of the structure and molecular binding. It exhibits cardioprotective activity in MI by potent antioxidant and anti-inflammatory properties. The presence of a phenolic hydroxyl group (-OH) is responsible for the strong antioxidant activity (Figure 8). The C-3 ketone group (C=O) and the extended aliphatic 10-chained hydrocarbon side chain add the lipophilic behavior, thus facilitating bioavailability and the intracellular signaling process [243].

### 7.6. SAR of Epigallocatechin Gallate

**Figure 9:**
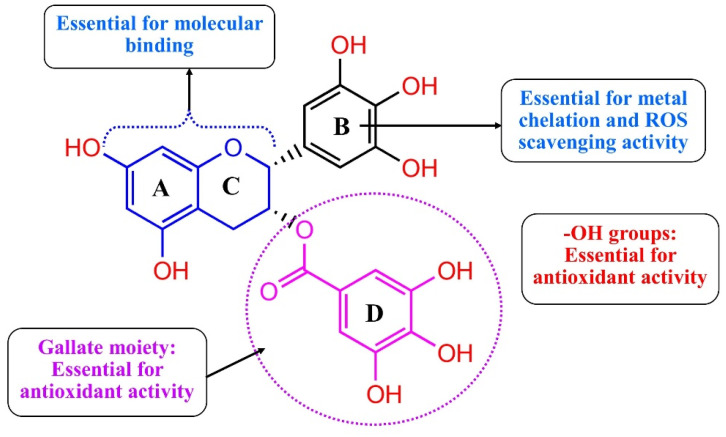
SAR of epigallocatechin gallate.

Epigallocatechin gallate, a 3-flavanol, is basically a catechin esterified with gallic acid. It contains a pyran ring, C, which is esterified at position 3 with gallate and is linked to three aromatic rings, A, B, and D. It shows strong cardioprotective activity in MI due to its unique polyphenolic structure with multiple –OH groups. The –OH groups and the gallate molecule are crucial for high antioxidant activity. The B-ring is essential for chelation of metal and excellent antioxidant activity (Figure 9). The A-ring and the attached pyran ring, C, are essential for molecular interaction with inflammatory signaling targets [244,245].

### 7.7. SAR of Scopoletin

**Figure 10:**
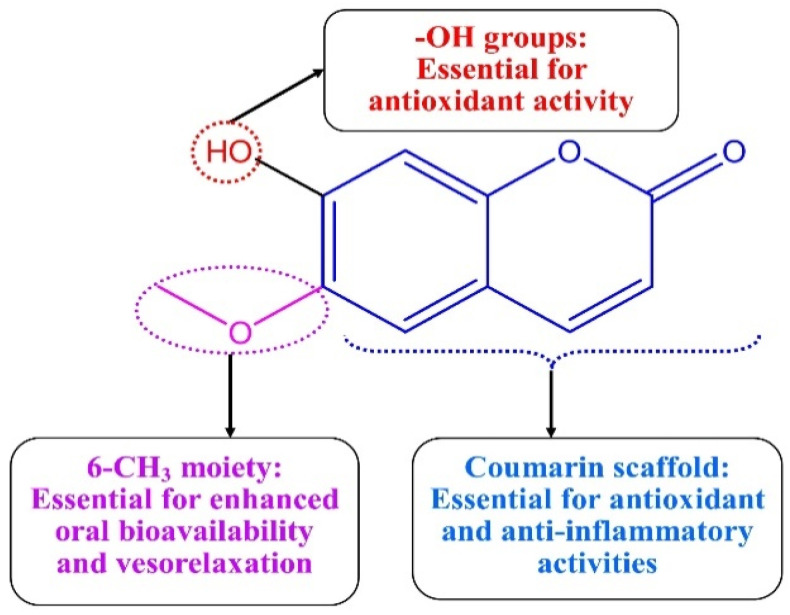
SAR of scopoletin.

Scopoletin, also known as 7-hydroxy-6-methoxycoumarin, is a natural coumarin derivative reported for cardioprotective properties in MI due to its anti-inflammatory, antioxidant, and vasorelaxant activities. It contains a core coumarin scaffold, which is key to showing antioxidant and anti-inflammatory activities. The 7-hydroxyl group (7-OH) is responsible for free radical scavenging and antioxidant activity. The 6-methoxy group (6-OCH_3_) may enhance oral absorption and bioavailability as well as vasorelaxation. Substitution on the aromatic ring produces different types of analogs (Figure 10). Any analog containing a nucleophilic moiety shows better antioxidant activity but easily gets metabolized. Analog with electrophiles shows opposite effects [246].

Since no direct SAR studies were conducted specifically for cardioprotective activity in MI, the proposed relationships between chemical structures and cardioprotective activities are inferred from the known antioxidant, anti-inflammatory, and pharmacological properties of the compounds reported in previous studies. Although the seven phytocompounds belong to distinct phytochemical classes, several common structural features may govern their cardioprotective effects in MI. The presence of phenolic -OH groups is a common characteristic contributing to antioxidant activity through free radical scavenging in combating MI. Compounds with a higher degree of hydroxylation, such as quercetin, EGCG, and ellagic acid, generally exhibit stronger antioxidant potential but often have poor bioavailability. Another important structural feature is the presence of conjugated aromatic systems, which facilitate electron delocalization and enhance interactions with molecular targets involved in oxidative stress, inflammation, and cardiac remodeling. Carbonyl-containing moieties, including the flavonol, isoflavone, lactone, and ketone structures, further contribute to target binding and biological activity. In contrast, methoxy substituents and hydrophobic side chains, as observed in biochanin A, scopoletin, and 10-gingerol, improve lipophilicity and membrane permeability, thereby enhancing pharmacokinetic properties. An optimal balance between hydroxylation, conjugation, and lipophilicity is critical for maximizing cardioprotective efficacy in MI, which requires more in-depth scientific investigations.

## 8. Clinical Trial

**Figure 11:**
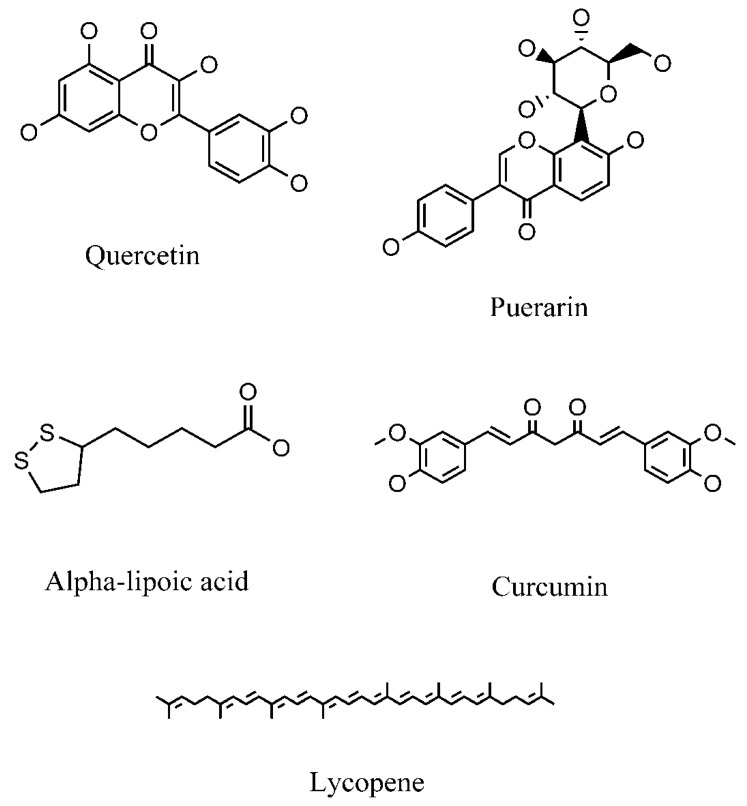
Plant-derived compounds under clinical trial for MI and oxidative stress management.

Numerous plant-derived compounds, which were reported to undergo clinical trials for the treatment of MI and oxidative stress, include quercetin, puerarine, curcumin, α-lipoic acid, and lycopene (Figure 11).

These clinical trials exhibited potential cardio-protective effects of these compounds. However, the weaknesses in their methods and the involvement of a small sample size do not yet allow these compounds to be translated into clinical use. Among them, quercetin underwent a multicenter randomized controlled trial where its cardioprotective potential was assessed in 143 ST-elevated MI patients [85]. Here, 73 patients were solely given standard medical therapy and the remaining 70 patients were infused with intravenous quercetin along with the standard therapy. The infarct size was measured by assessing CK-MB (creatinine kinase–myocardial band) area under the curve, whereas intramyocardial hemorrhage-related bleeding was measured by conducting MRI scans of the heart. This study reported that quercetin is able to significantly decrease the infarct size and prevent the reperfusion-associated intramyocardial hemorrhage when administered intravenously along with standard therapy. However, one downside of the study was that it mainly focused on the immediate clinical outcomes and did not keep track of long-term patient data. Having stated that, the study results showed a possibility of turning quercetin into an adjunct treatment option in managing acute MI. Nevertheless, quercetin must be tested further in large-scale clinical trials with long-term patient follow-up before it can be considered as an adjunct treatment option.

In another clinical trial, puerarin was investigated to examine its effectiveness compared with granulocyte colony-stimulating factor (G-CSF) in treating acute MI [100]. This comparative study was carried out with 79 patients having anterior AMI. During the study, subjects were split into three groups, where one group was regarded as the control and received standard treatment alone. Out of the two remaining groups, one group was parenterally administered with puerarin along with standard therapy, while the other group was parenterally administered with G-CSF combined with standard therapy. The study concluded that puerarin is not only capable of reducing the infarct size but also capable of reducing inflammatory biomarkers, including MMP-9, IL-6 and TNF-alpha. Similar to the quercetin trial, in this study, the infarct size was measured by assessing the CK-MB area under the curve. Though G-CSF also reduced the infarct size, the inflammatory biomarkers were elevated in that group. This study involved a limited number of patients and did not conduct prolonged follow-up for the long-term clinical outcomes despite encouraging findings. Hence, large-scale double-blind placebo-controlled randomized trials are required to further prove its potential to be used clinically.

Moreover, to check if curcumin supplementation combined with piperine is able to mitigate myocardial injury succeeding acute MI, a randomized double-blind clinical trial was conducted. The trial included 72 acute MI patients, and they were categorized into two groups, one of which was called the placebo group, with 34 patients. The other group was known as the curcumin group, including 38 patients. While the curcumin group was given 500 mg curcumin combined with piperine daily to facilitate curcumin absorption, the placebo group received nothing but the placebo during the 8-week study period. The study stated that supplementation with curcumin markedly diminishes HbA1C, LDL, ALT, and ALP, while increasing HDL levels in acute MI patients [247]. This finding demonstrates the metabolic and hepatoprotective benefits of curcumin in post-MI patients. However, no influence was noticed on ejection fraction and cardiac troponin 1 level, which implies that curcumin has no direct role in post-myocardial infarction-related recovery.

Also, 112 post-non-Q MI patients who had type 2 diabetes mellitus went through a separate randomized open-label clinical trial where 59 patients considered as the main group were administered 600 mg α-lipoic acid orally on a daily basis for a duration of 4 months in addition to oral antidiabetic medication and basic cardiovascular drugs [248]. The rest of the 53 patients were categorized as the experimental patient group and were given baseline therapy alone. This study also included a control group consisting of nearly healthy 40 people. The trial observed a reduction in CRP, IL-6, and TNF-α, except IL-10, in the main group. Therefore, the investigation preliminarily supported the use of α-lipoic acid for the above-mentioned patients due to its antioxidant potential, along with vasorelaxation and anti-inflammatory properties. The trial did not conduct any experiment to assess cardiovascular events, though.

Furthermore, a clinical trial involving lycopene-enriched tomatoes was carried out in a healthy population [249]. The trial came to the conclusion that consumption of lycopene-enriched tomatoes could reduce postprandial oxidative stress and inflammatory response. However, this trial did not involve any MI patients. In another clinical trial, which was a single-center, two-arm, placebo-controlled study, the impact of lycopene was observed on cardiac biomarkers, including CKMB, troponin 1, and high-sensitive CRP, among patients undergoing PCI [177]. The study included 45 patients, and they were divided into two groups, namely the treatment group comprising 23 patients and the control group comprising 22 patients. While the control group was given only standard therapy following PCI, the treatment group was provided with oral lycopene tablets before and after PCI. The study reported that the treatment group exhibited significant prevention of CKMB rise following PCI. However, it did not show any significant alteration of troponin 1 and highly sensitive CRP in comparison to the control group. Although in this trial, lycopene was seen to prevent post-PCI-related cardiac muscle damage, it did not include MI patients directly. Having stated that, this trial showed initial clinical evidence for conducting future clinical trials involving lycopene in post-MI patients.

To summarize, although some compounds demonstrated promising results in preliminary clinical trials, these studies were largely based on small studies, short-term outcomes, and limited follow-up. Hence, these evidences are not yet sufficient to support their therapeutic use in MI. Therefore, further large-scale clinical studies are required to assess their clinical applicability.

## 9. AI-Assisted Drug Discovery and Translational Perspectives

The development and discovery of drugs generally take more than a decade and cost an average of US$2.8 billion, but almost 90% of drug candidates do not make it through clinical development or regulatory review. Chemical space contains > 10^60^ possible molecules, but conventional screening and optimization approaches are slow and resource-intensive. Artificial intelligence (AI) and other in silico approaches have transformed this process by enabling rapid identification of hit and lead compounds, efficient target validation, and rational drug design [250]. Therefore, AI-assisted drug discovery has emerged as a valuable strategy for accelerating the development of therapeutically promising compounds while reducing the costs and time requirements [251].

### 9.1. ML/QSAR-Based SAR Prediction

Quantitative structure–activity relationship (QSAR) modeling is a computational technique that enables the prediction of biological activities, including antioxidant and cardioprotective effects, from molecular descriptors derived from the chemical structures of compounds [252]. QSAR models are particularly useful for the identification and screening of bioactive phytochemicals because they predict biological properties of new and untested compounds belonging to the same chemical class based on certain structural features. Also, QSAR enables the study of the biochemical interactions of complex phytochemicals with biological targets such as enzymes and receptors. Recent developments in machine learning have greatly improved the predictive capabilities of QSAR methods [253]. In a typical QSAR model, supervised machine learning algorithms are applied and trained on high-quality experimental datasets to correlate molecular descriptors to biological responses. Modern deep learning-based or descriptor-free QSAR methods, on the other hand, can utilize large datasets to automatically learn complex structure–activity patterns, speeding up lead identification, virtual screening, and rational drug design even for yet-to-be-synthesized compounds [254]. The QSAR models can be used to quantitatively assess the influence of structural features such as the number and position of hydroxyl groups, methoxy substitutions, glycosylation patterns, conjugated double bonds and planarity on the antioxidant potency and cardioprotective activity of the antioxidant phytochemicals discussed throughout this review. QSAR analysis can predict if structural modifications of flavonoids, phenolic acids, stilbenes or terpenoids are likely to improve free radical scavenging capacity and interactions with oxidative stress-related targets.

### 9.2. Network Pharmacology Approaches

Network pharmacology has become a potent tool in modern drug discovery, bridging systems medicine with computer and information sciences to probe the complex relationships between drugs, biological targets, and diseases. This integrative approach constructs interconnected protein–compound and disease–gene networks to illuminate the molecular mechanisms underlying the therapeutic effects of multi-component treatments, especially those from medicinal plants [255]. Network pharmacology shifts focus from the classical single-target drug model to a network-based therapeutic frame, offering a more holistic understanding of disease intervention. Moreover, the application of network topology analyses (degree, betweenness, centrality, and modularity) enables the identification of bioactive compounds and core molecular targets, allowing a complete understanding of drug action mechanisms and therapeutic pathways [256]. Network pharmacology provides a systems-level perspective for understanding the antioxidant protection against MI. By constructing the compound–target–disease network, it can identify the key molecular targets and signaling pathways of oxidative stress, inflammation, apoptosis and cardiac remodeling. This approach can contribute to the elucidation of the multi-target mechanisms of antioxidant compounds and facilitate the discovery of novel cardioprotective agents for MI treatment. Many antioxidants of plant origin exert cardioprotective effects via multiple molecular targets, making network pharmacology particularly suitable to elucidate their mechanisms of action. Flavonoids, phenolic acids, terpenoids and alkaloids reviewed herein have the potential to simultaneously regulate oxidative stress, inflammation, apoptosis, mitochondrial dysfunction and fibrosis-related signaling.

### 9.3. Molecular Docking and Virtual Screening

Virtual screening is a computational approach for drug discovery, which predicts the interaction of compounds from large chemical libraries with biological targets and, therefore, helps to identify potential drug candidates. This greatly reduces the time, cost and resources required for experimental screening by ruling out unlikely active compounds prior to lab testing [257]. Molecular docking, a key structure-based virtual screening technique, predicts the binding affinity and interaction modes between ligands and target proteins. Various docking software, including AutoDock, AutoDock Vina, GOLD, Glide, MOE, ICM, and FlexX, are commonly employed, with the choice depending on the research objectives and computational resources available [258]. Molecular docking is a useful in silico tool for screening of potential antioxidant lead compounds against myocardial infarction by predicting their binding affinity with key proteins implicated in oxidative stress and cardiac injury. It guides the prioritization of phytochemicals with strong and stable interactions with relevant targets and potential cardioprotective effects. This strategy reduces experimental workload and accelerates the selection of promising antioxidant candidates for further validation in MI therapy. Molecular docking and virtual screening can rank the phytochemicals discussed in this review according to their predicted interactions with myocardial infarction-related proteins. Flavonoids (quercetin or luteolin), phenolic acids (gallic acid or ferulic acid) and terpenoids (ursolic acid) can be screened computationally against relevant targets before in vitro and in vivo evaluation. Docking combined with molecular dynamics simulations and MM/PBSA calculations could further enhance the prediction of binding stability and help identify the most promising antioxidant leads.

### 9.4. Multi-Omics Integration for Target Identification

As modern medical science progresses, the knowledge of the complex mechanisms underlying disease becomes increasingly important. Omics-based technologies provide high-throughput methods that generate comprehensive biological information about human diseases rapidly. These include genomics, transcriptomics, proteomics, metabolomics, single-cell transcriptomics, and spatial and integrative multi-omics platforms. All omics technologies identify alterations in molecules linked to the disease, such as differentially expressed genes, proteins and metabolites. These changes can be used as biomarkers and can help distinguish between disease and normal biological states by highlighting disrupted pathways [259]. Significantly, the multi-omics combination can greatly enhance the research of myocardial infarction (MI) by clarifying the mechanism of antioxidant compounds in terms of protective effects in various biological strata, such as the modulation of oxidative stress, inflammatory signaling, and energy metabolism, thus supplying a panoramic view of their cardio-protective potential [260,261]. Multi-omics approaches may be able to elucidate the different cardioprotective efficacy of related phytochemicals. Linking structural features of flavonoids, polyphenols, terpenoids, and alkaloids to modulation of oxidative stress, mitochondrial function, immune responses, and myocardial remodeling can be achieved by integrating transcriptomics, proteomics, metabolomics, and single-cell sequencing data with phytochemical intervention studies.

### 9.5. Generative AI for Phytochemical Derivative Optimization

Generative deep learning models have emerged as powerful tools for the de novo design and optimization of phytochemical derivatives in the modern drug discovery pipeline. Architectures like variational autoencoders (VAEs), generative adversarial networks (GANs) and transformer-based molecular generators (e.g., MolGPT and related DrugGPT frameworks) are capable of learning latent chemical representations and generating new structures with better pharmacological properties. In the context of this review, the structure–activity relationship (SAR) patterns identified for bioactive phytochemicals can be used as constraints to guide these generative models towards chemically valid and biologically relevant space, thus increasing the chances of producing lead-like candidates [262,263]. Together, these generative AI approaches offer a way for rational phytochemical scaffold optimization by integrating SAR knowledge and learned chemical representations, and accelerate the discovery of optimized antioxidant derivatives with potential therapeutic relevance in myocardial infarction and other oxidative stress-related diseases. The SAR patterns summarized throughout this review provide valuable knowledge for AI-guided molecular optimization. Structural characteristics associated with enhanced antioxidant activity, including catechol moieties, hydroxyl substitution patterns, conjugated aromatic systems, and lipophilic modifications that improve membrane permeability, can serve as design constraints for generative AI models. These models may propose novel derivatives of flavonoids, phenolic acids, lignans, or terpenoids with improved antioxidant potency, metabolic stability, oral bioavailability, and target selectivity while preserving favorable phytochemical scaffolds.

## 10. Conclusions

This review summarizes the evidence that confirms the contribution of oxidative stress in the pathogenesis and progression of myocardial infarction. It also sheds light on the underlying cardio-protective mechanisms of bioactive 51 natural antioxidant compounds, along with the reported SARs of 7 of them. Considering the vast number of naturally occurring bioactive antioxidant compounds, the number of antioxidant compounds that have been investigated to treat myocardial infarction is comparable to the tip of the iceberg. The SAR regarding the cardio-protective activity of these compounds reported is even too little. Although plenty of researchers have focused on finding the potential cardio-protective activity of natural antioxidant compounds against MI, the overwhelming majority of them have used animal models. Moreover, a single, naturally occurring, unmodified antioxidant compound has not fulfilled all the requirements to be a novel drug to date. Therefore, this review recommends the continuation of more work regarding the structural modification of the already explored compounds to find a more potent and safer drug for the purpose of bringing them from laboratory to bedside. To facilitate the structural modification, this review also suggests the conduction of newer works exploring the SAR of natural antioxidant compounds with a focus on their development and formulation as drugs.

## Data Availability

No new data were created or analyzed in this study. Data sharing is not applicable to this article.
